# Pain management in complex wounds: a scoping review and analysis using comfort theory

**DOI:** 10.1590/1980-220X-REEUSP-2025-0439en

**Published:** 2026-02-27

**Authors:** Mariana Érica da Silva Paixão, Yzis Oliveira Pontes Pereira, Amanda Meireles Medeiros, Rute Xavier Silva, Vívian Lopes Miele, Jocelly de Araújo Ferreira, Oriana Deyze Correia Paiva Leadebal

**Affiliations:** 1 Universidade Federal da Paraíba, Departamento de Enfermagem Clínica, João Pessoa, PB, Brazil.

**Keywords:** Pain Management, Wounds and Injuries, Chronic Pain, Clinical Protocols, Analgesia

## Abstract

**Objective:**

To map, from the perspective of Kolcaba’s Comfort Theory, scientific evidence of interventions for pain management in people with complex wounds.

**Methods:**

Scoping review conducted across seven databases and grey literature. The application *Rayyan* was used for material selection. Data processing was performed using the IRaMuTeQ software, employing Descending Hierarchical Classification, based on the topic sample results.

**Results:**

The sample, consisting of 27 publications, comprised the text *corpus*. Interventions for pain management in people with complex wounds encompassed physical and psycho-spiritual dimensions of Comfort Theory, with a prevalence of ibuprofen-impregnated dressings, technologies such as virtual reality, eye tracking, and diode laser, as well as pharmacotherapies and complementary approaches, such as relaxation techniques, music therapy, and cognitive-behavioral therapy.

**Conclusion:**

This review mapped interventions in pain management for complex wounds, highlighting benefits such as immediate relief, improved quality of life, and promotion of healing, in line with Kolcaba’s Comfort Theory.

## INTRODUCTION

Complex wounds are defined as any loss of continuity in the anatomical structure of the skin or deeper tissues. They do not show adequate progression in the healing process and, as such, persist for six weeks or more, despite active therapeutic interventions. In these wounds, ineffective tissue repair mechanisms occur, posing a challenge for clinical management. It is known that some comorbid conditions, such as vasculopathies, neuropathies, neoplasms, physical immobility, and nutritional deficiencies, contribute to the chronicity of injuries^(1,2,3)^.

Prolonged healing time and the presence of comorbidities that compromise cell regeneration contribute to the increased complexity of these wounds. As a result, there are impacts on the physical, emotional, and social spheres of those affected, compromising their quality of life. Furthermore, prolonged and continuous treatments are required, generating significant costs for healthcare systems^(2,4)^.

The prevalence of complex wounds in the world population is not yet fully known due to multiple factors contributing to the occurrence of this comorbidity; however, it is estimated that 20 million people globally suffer from these lesions, of which 5 million are Brazilian^(5)^.

From a pathophysiological point of view, it is known that the occurrence of complex wounds is frequently related to underlying chronic conditions and alterations in tissue perfusion. Current literature indicates that the most prevalent types are pressure injuries (PI), vascular ulcers (arterial and venous), diabetic ulcers, and surgical wounds that heal by secondary intention. These etiologies, although distinct in their origin, generate clinical challenges for promoting wound healing in contexts of systemic and local involvement, requiring individualized and multidisciplinary therapeutic approaches, with a clear leading role for Nursing^(6,7)^.

Moreover, clinical care for people with complex wounds involves managing various needs associated with living with a wound, including pain management, a multifactorial phenomenon encompassing sensory, emotional, and social dimensions, which makes its management challenging^(6)^.

The origin of complex wounds is not limited to the pathophysiological process of the wound, being related to comorbidities such as vascular diseases, Diabetes Mellitus, Systemic Arterial Hypertension and neuropathies, which intensify pain sensations. These painful stimuli are exacerbated during the cleaning process due to the high sensitivity of the affected area and the mechanical interventions involved, such as removal of the primary dressing, cleaning, debridement of non-viable tissues, and application of a new dressing. These procedures, while essential to treatment, are often described as moments of significant painful experiences^(6,8)^.

Considering the multifactorial and subjective context, it is important to highlight that the goal of nursing care for the clinical management of pain will not always be analgesia, given the chronic nature of wounds. At a minimum, it may involve promoting comfort, defined by Katharine Kolcaba as an immediate experience of empowerment based on needs for relief, tranquility, or transcendence, which are met in physical (referring to bodily sensations), psycho-spiritual (meaning of life/spirituality, which includes self-esteem, self-concept), environmental (related to environmental elements), and sociocultural (involving interpersonal, family, social, and financial relationships) contexts^(9,10)^.

The literature includes reviews describing pharmacological and non-pharmacological interventions for pain management in the context of complex wounds, with advances in technologies, analgesic strategies, and complementary approaches such as relaxation techniques, music therapy, and cognitive-behavioral therapy^(11,12,13,14)^. However, these studies, while relevant, have a predominantly clinical and biomedical focus, without organizing care from a broader perspective that considers the dimensions of patient comfort.

Therefore, a research gap is observed that justifies the development and systematization of interventions based on theoretical nursing frameworks, especially Kolcaba’s Comfort Theory. This framework, by understanding comfort as a measurable outcome of care and as an instrument that guides the nursing work process, allows for a systematized, holistic, and patient-centered approach.

Based on these considerations, the present study aimed to map the scientific evidence of interventions for pain management in people with complex wounds, from the perspective of Kolcaba’s comfort theory.

## METHOD

### Design of Study

A scoping review study was developed, conducted in accordance with JBI’s methodological guidelines for scoping reviews and as recommended by the guideline *Preferred Reporting Items for Systematic Reviews and Meta-Analyses extension for Scoping Reviews* (PRISMA-ScR)^(15,16)^. The review protocol was registered in the *Open Science Framework* (OSF) with DOI 10.17605/OSF.IO/Q3B25 to make the study information public, encourage transparency, and enable the research search strategy to be replicated.

The review was developed in the following stages: identification of the research question, identification of relevant studies, study selection, data mapping, data collection, summarization, and reporting of results^(17)^.

### Research Question

The mnemonic strategy Population, Concept, and Context (PCC) was used, where Population – Adults with complex wounds, Concept – interventions performed for pain management, and Context – healthcare service, which guided the development of the review guiding question: “What scientific evidence is available in the literature on pain management interventions in people with complex wounds, developed in healthcare settings?” The search was conducted from december 2024 to april 2025.

### Eligibility Criteria

For the selection of the material comprising the textual corpus, the following inclusion criteria were adopted: complete original articles, made available in full and free of charge in electronic format (CAFe access); dissertations and theses, without language restrictions and without time limitations. The following were excluded: letters to the editor and letters to the reader, abstracts in conference proceedings, incomplete articles, studies in the project phase, or studies without results yet.

### Search Strategy

A search was conducted in the databases *National Library of Medicine and National Institutes of Health* (PUBMED), *Web of Science* (WOS), *Excerpta Medica* (Embase), *Cumulative Index to Nursing and Allied Health Literature* (CINAHL), SCOPUS (Elsevier), *Cochrane Library*, Latin American and Caribbean Literature in Health Sciences (LILACS), accessed through the Federated Academic Community (CAFe), using terms found in *Medical Subject Headings* (MeSH) and Health Sciences Descriptors (DeCS). The review also covered the unconventional literature (grey literature) in the repository *ProQuest Dissertations and Thesis Global*, Brazilian Digital Library of Theses and Dissertations (BDTD) and Google Scholar (limited to the first 100 results).

The keywords used were ‘Manejo da Dor’, ‘Ferimentos e Lesões’, ‘Dor Crônica’, ‘Protocolos Clínicos’ and ‘Analgesia’. To increase the search sensitivity, these terms were combined with their corresponding translations, such as ‘*Pain Management*’, *Wounds and Injuries*’, *Chronic Pain*’, ‘*Clinical Protocols*’ and ‘*Analgesia.* The Boolean operators OR and were used. A specific search strategy was developed for each database, taking into account its particularities, with the aim of optimizing the retrieval of relevant information as described in Chart 1.

**Chart 1:** Search strategies used in the databases – João Pessoa, PB, Brazil, 2025.

Database	Search strategy
MEDLINE/PubMed	(“Pain Management”[MeSH Terms] OR “Pain Management”[Title/Abstract]) AND (“Wounds and Injuries”[MeSH Terms] OR “Wounds and Injuries”[Title/Abstract]) AND (“Chronic Pain”[MeSH Terms] OR “Chronic Pain”[Title/Abstract])
Embase	(‘complex wounds’ OR ‘chronic wounds’:ti,ab,kw OR ‘chronic wounds’) AND (‘pain management’:ti,ab,kw OR ‘pain management’) AND (‘analgesia’:ti,ab,kw OR ‘analgesia’)
SCOPUS	(TITLE-ABS-KEY (“Complex Wounds”) OR TITLE-ABS-KEY (“Wounds and Injuries”) AND TITLE-ABS-KEY (“Pain Management”) AND TITLE-ABS-KEY (“Chronic Pain” ) )
*Web of Science*	TS = (“Pain management”) AND TS = (“Wounds and injuries”) OR TS = (“Complex Wounds”) OR TS = (“Chronic Wounds”) AND TS = (“Chronic Pain”) AND TS = (“Clinical Protocols”) OR TS = (“Treatment Protocols”)
LILACS	(“Manejo da Dor”) OR (“Manejo del Dolor” ) AND (“Ferida” ) OR (“Herida”) OR (“Feridas”) OR (“Heridas”) AND (“Dor Crônica”) OR (“Dolor Crónico”) AND (“Cuidados de Enfermagem”) OR (“Atención de Enfermería”) OR (“Assistência de Enfermagem”) OR (“Cuidado de Enfermería”) AND (instance:”regional”)
*Cochrane Library*	“Pain Management” in Title Abstract Keyword AND “Wounds and Injuries” in Title Abstract Keyword OR “Complex Wounds” in Title Abstract Keyword OR Injury in Title Abstract Keyword AND “Chronic Pain” in Title Abstract Keyword
CINAHL	“Pain Management” AND ( “Wounds and Injuries” ) OR Injury AND “Chronic Pain” AND “Clinical Protocols”
Biblioteca Digital Brasileira de Teses e Dissertações	Feridas AND “Manejo da Dor”
ProQuest	(“Complex Wounds” OR “Chronic Wounds”) AND “Chronic Pain”
Google Scholar	(“Complex Wounds” OR “Chronic Wounds” OR “Wounds and Injuries” OR Injury) AND “Pain Management” AND “Chronic Pain” OR “Analgesia”

Source: Research data, 2025.

### Selection of Studies

After searching the databases, all records found were exported to the Zotero reference manager, version 7.0.10, for duplicate removal. The selection and evaluation of studies was carried out independently and blindly by two reviewers, with a third reviewer consulted in cases of disagreement, using the web application web *Rayyan*. The pre-selection of studies was carried out by reading the title and abstracts, based on the inclusion and exclusion criteria. The pre-selected studies were then fully read for definition of the eligible ones.

Finally, a search was conducted in the reference lists of the included studies to identify other articles not located in previous searches. The studies deemed eligible in this phase were included with the others previously selected, and formed part of the scoping review *corpus*. The selection process is presented according to the PRISMA 2020 flow diagram.

### Data Extraction

For extracting information from the *corpus,* an instrument developed by the researchers was used, structured in a Microsoft Excel spreadsheet, with the following variables: database, journal, year of publication, authorship, language, DOI, title, country of origin, objective, methodological design, main results, and conclusion.

### Presentation of the Data

A synthesis of the contents of each study was prepared, giving rise to the textual *corpus*, which in turn was entered in the software *Interface de R pour les Analyses Multidimensionnelles de Textes et de Questionnaires* (IRaMuTeQ) version 0.7 alpha2, through multivariate analysis of Descending Hierarchical Classification (DHC), which allowed the grouping of similar vocabularies into classes of textual segments and the presentation of semantic relationships through a dendrogram generated by the software^(18)^.

Following the presentation of the DHC proposed by the software, the classes (5 classes) were reorganized according to content analysis based on the theoretical framework by Katharine Kolcaba (2003), which allowed the identification of 2 categories.

### Ethical Aspects

This scoping review used exclusively publicly available secondary data and therefore did not require approval from a Research Ethics Committee. All stages were conducted in accordance with ethical principles and applicable methodological guidelines, ensuring respect for the copyright of the productions analyzed.

## RESULTS

**Figure 1:**
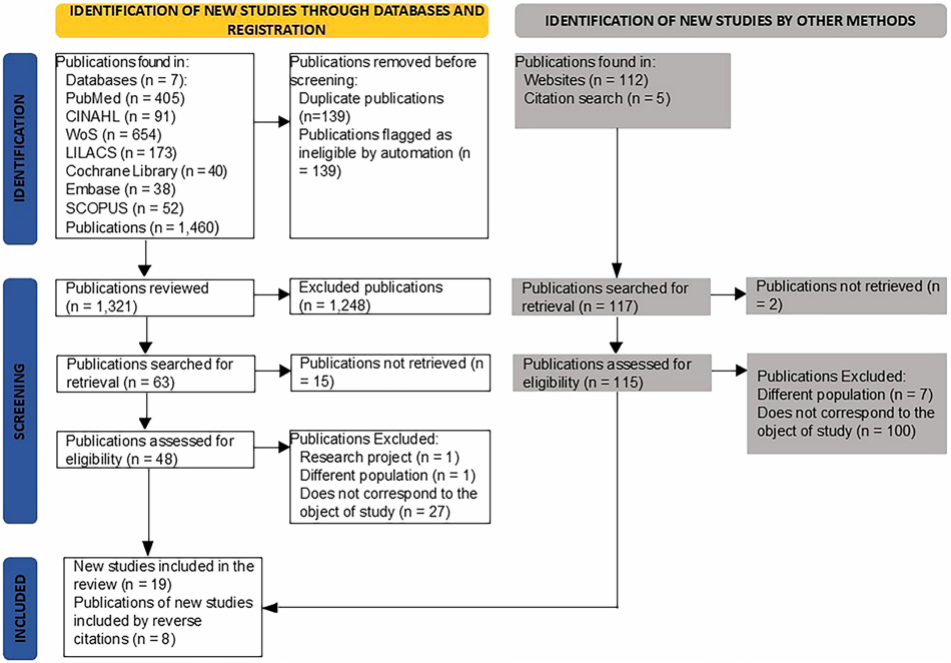
PRISMA-ScR flowchart of study selection and inclusion process in the scoping review. João Pessoa, PB, Brazil, 2025.

The textual *corpus* of the study resulted from 27 articles out of the 1,577 found in databases and repositories, whose identification, screening, and eligibility process occurred as shown in Figure 1.

To facilitate the presentation, the most relevant data characterizing the articles were organized in a chart (Chart 2).

**Chart 2:** Characterization of the studies – João Pessoa, PB, Brazil, 2025.

ID*	Author/Year	Title	Objective
A1	Bechert and Abraham, 2009^(13)^	Pain management and wound care	To discuss the etiology of pain, as well as to provide the reader with strategies for managing painful wounds once they have been identified.
A2	Ffrench et al., 2023^(19)^	Systematic review of topical interventions for the management of pain in chronic wounds	To establish a robust understanding of the level of effectiveness of available topical interventions for the treatment of wound-related pain in individuals with lived experience of chronic wounds.
A3	Guglielmo, 2024^(20)^	The ‘Surrounding the Dragon’ (Wei Ci) Acupuncture Technique: A Systematic Review	To determine the evidence-based effectiveness of the ancient Traditional Chinese Medicine (TCM) acupuncture technique ‘Surrounding the Dragon’.
A4	Healy et al., 2023^(21)^	Chronic wound-related pain, wound healing and the therapeutic potential of cannabinoids and endocannabinoid system modulation	To comprehensively summarize the findings to date on the potential of cannabinoids, endocannabinoid system modulators, and cannabis-based medicines for the treatment of chronic wound-related pain and wound healing.
A5	Mervis and Federman, 2018^(11)^	Pain Management in Patients with Chronic Wounds	To provide an evidence-based framework for the pharmacological and non-pharmacological management of pain in patients with chronic wounds.
A6	Metcalfe, 2015^(22)^	Pain management in palliative patients with chronic wounds	To define pain in relation to chronic palliative wounds, examine the incidence and rates of chronic pain in this patient group, and review the literature for the best management strategies.
A7	Price et al., 2007^(12)^	Managing painful chronic wounds: the Wound Pain Management Model	To assess wound pain and treatment strategies, focusing on persistent chronic pain and pain associated with local wound treatment, including dressing changes.
A8	Richardson, 2012^(23)^	An introduction to the biopsychosocial complexities of managing wound pain	Discuss the treatment of acute and chronic wounds, focusing on the specific skills needed to manage pain associated with different types of wounds and addressing important areas for dressing changes.
A9	Woo et al., 2013^(24)^	Evidence-based approach to manage persistent wound-related pain	To provide an evidence-based approach to the treatment of persistent pain in people with chronic wounds.
A10	Arapoglou et al., 2011^(25)^	Analgesic efficacy of ibuprofen-releasing foam dressing compared with local best practice for painful exuding wounds	To examine whether the etiology of the wound affects the analgesic properties of an ibuprofen-releasing foam dressing, which has previously been shown to reduce pain in wounds of various etiologies, compared with local best practices (LBP).
A11	Araújo et al., 2021^(8)^	Realidade virtual durante o manejo da dor em pessoas com feridas crônicas: estudo experimental	To evaluate the use of virtual reality in immersive environments and realistic simulations for pain relief in patients with chronic wounds during dressing changes.
A12	Gottrup et al., 2007^(26)^	Less pain with Biatain–Ibu: initial findings from a randomised, controlled, doubleblind clinical investigation on painful venous leg ulcers	To investigate whether ibuprofen foam dressings relieve venous ulcer pain without compromising the beneficial properties of moist wound healing, with an acceptable safety profile.
A13	Humbert et al., 2013^(27)^	Efficacy and safety of a gauze pad containing hyaluronic acid in treatment of leg ulcers of venous or mixed origin: a double-blind, randomized, controlled trial	To investigate the efficacy and safety of a gauze compress containing hyaluronic acid (HA) in the local treatment of venous leg ulcers, compared to its neutral vehicle.
A14	Pittler and Ernst, 2012^(28)^	Horse chestnut seed extract for chronic venous insufficiency	To review the efficacy and safety of horse chestnut seed extract (HCSE) versus placebo or reference therapy for the treatment of CVI.
A15	Romanelli et al., 2009^(29)^	Ibuprofen slow-release foam dressing reduces wound pain in painful exuding wounds: preliminary findings from an international real-life study	To compare the effect of an ibuprofen-releasing foam dressing (Biatain Ibu®) with best local practices in the treatment of painful exudative wounds.
A16	Kitala et al., 2022^(30)^	Eye-tracked computer games as a method for pain perception alleviation in chronic wound management	To evaluate the usability of eye-tracking games that distract the patient during wound care activities in an outpatient surgical clinic and, therefore, to verify whether it is worthwhile to introduce eye trackers into the daily clinical routine.
A17	Rook et al., 2019^(31)^	Mepilex® Border Flex — results of an observational study in German specialist wound care centres	To evaluate the treatment of a variety of exudative wound types with a selfadhesive silicone-coated foam dressing.
A18	Nair, 2018^(32)^	Microcurrent as an adjunct therapy to accelerate chronic wound healing and reduce patient pain	To evaluate the effectiveness of microcurrent as an adjunct therapy in reducing wound size and pain score in patients with chronic wounds.
A19	Swift et al., 2024^(33)^	Taking the pain out of wound healing with microcurrent electrical stimulation therapy	To evaluate the use of Accel-Heal Solo in reducing pain associated with slow-healing wounds and its impact on analgesic dependence.
A20	Tang et al., 2023^(34)^	The associations between diode laser (810 nm) therapy and chronic wound healing and pain relief: Light into the chronic wound patient’s life	To determine the associations between diode laser therapy and chronic wound healing or pain relief.
A21	Acevedo, 2020^(35)^	Successful treatment of painful chronic wounds with amniotic and umbilical cord tissue: A case series	It does not have any.
A22	Bradbury et al., 2008^(36)^	Measuring outcomes with complex patients: an audit of the effect of Actiform Cool on painful wounds	To explore the effect of Actiform Cool on wound-related pain.
A23	Flanagan et al., 2006^(37)^	Case series investigating the experience of pain in patients with chronic venous leg ulcers treated with a foam dressing releasing ibuprofen	To profile and characterize individuals using ibuprofen foam dressing.
A24	Freedman et al., 2004^(38)^	Practical treatment of pain in patients with chronic wounds: pathogenesis-guided management	To develop a comprehensive pain management proposal based on addressing the pathological causes of chronic wounds.
A25	Hampton, 2007^(39)^	Chronic pain in wounds: a report on 11 case studies	To investigate the effectiveness of ibuprofen-impregnated dressings in reducing pain and their impact on quality of life.
A26	Maida and Corban, 2017^(40)^	Topical Medical Cannabis: A New Treatment for Wound Pain—Three Cases of Pyoderma Gangrenosum	To evaluate the efficacy and safety of treatment with topical medicinal cannabis in non-genetically modified organic sunflower oil in patients with pyoderma gangrenosum.
A27	Eriksson et al., 2021^(41)^	Chronic wounds: Treatment consensus	To formulate an unbiased consensus on the best treatment for chronic wounds.

Source: Prepared by the authors, 2025.

*A: article, followed by the sequential number.

As observed in Chart 2, the studies were published between 2004 and 2024, with the highest occurrence of publications in the years 2023, with three studies (11.2%), and in 2024, with two studies (7.40%). A similar frequency of two studies (7.40%) was also observed in the years 2020, 2018, 2013, 2012, 2009, 2008, and 2007, while the years 2022, 2021, 2019, 2017, 2015, 2011, 2006, and 2004 presented only one study each (3.70%).

Regarding countries of origin, the United Kingdom stood out with six (22.3%), followed by five (18.5%) from the United States of America. Canada, China, Denmark, and Ireland each contributed with two studies (7.40%). Brazil, Malaysia, Italy, Greece, France, Poland, Germany, and England each contributed with one study (3.70%).

Regarding the levels of evidence, nine (33.3%) were level I, six (22.2%) were level II, two (7.40%) were level III, three (11.1%) were level V, six (22.2%) were level VI, and finally, one (3.70%) was level VII. Regarding the language, all the articles were in English.

**Figure 2:**
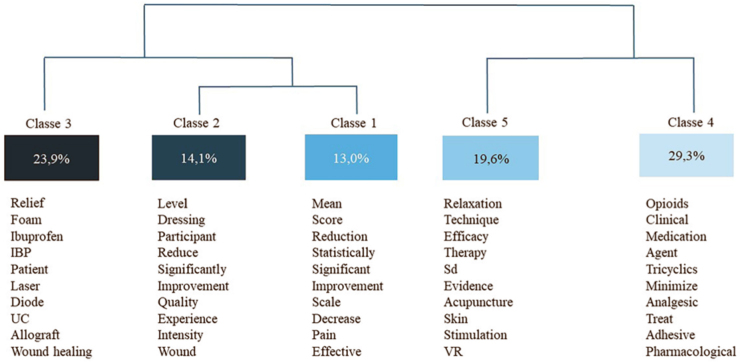
Descending Hierarchical Classification Dendrogram of the Text *Corpus* processed by IRaMuTeQ software. João Pessoa, PB, Brazil, 2025.

Figure 2 illustrates the dendrogram resulting from the DHC analysis, consisting of 5 classes named according to their content expression, and originating from the text *corpus*, consisting of 27 texts, 116 textual segments, and 4,072 words, with a utilization rate of 79.31% of the processed segments, considered a good utilization percentage.

**Figure 3:**
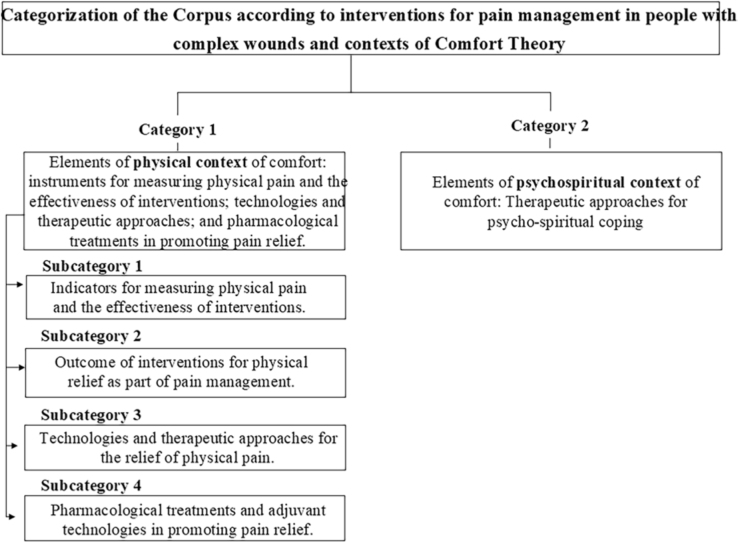
Categorization of the Corpus according to interventions for pain management in people with complex wounds, based on the contexts of Comfort Theory. João Pessoa, PB, Brazil, 2025.

Following the analytical exercise, supported by the theoretical framework by Katharine Kolcaba^(10)^ about this text *corpus* categorized by *software* IRaMuTeQ, 2 categories and 4 subcategories emerged. The identified categories represent physical and psycho-spiritual interventions for pain management in complex wounds, which are presented below in Figure 3.

### Category 1: Elements of the physical context of comfort: instruments for measuring physical pain and the effectiveness of interventions; technologies and therapeutic approaches; and pharmacological treatments in promoting pain relief.

In category 1, the physical dimension of the Comfort Theory stood out, which recommends the use of instruments, interventions, and technologies that allow measuring and alleviating physical pain, favoring more effective therapeutic responses in the care of people with complex wounds.

#### *Subcategory 1* – **Instruments for measuring physical pain and the effectiveness of interventions**

Subcategory 1 demonstrates the measurement instruments and their effectiveness in pain management interventions, such as the use of standardized scales like the Visual Analogue Scale (VAS), which allows for an objective assessment of pain before and after the implementation of interventions, by allowing the verification of whether there has been a statistically significant reduction in pain symptoms^(21,22,25,26,27,28,29,31,32,35,36,40)^.

#### *Subcategory 2* – **Outcome of interventions for physical relief as part of pain management**

Subcategory 2 reflects the results of direct physical wound care interventions as part of pain management. Following the clinical actions, the terms related to the results were: reduce, smaller, low, improvement, quality, and decrease. These words reflect the effectiveness of strategies, such as the use of gauze dressings containing hyaluronic acid (HA), which has been shown to have a favorable impact on wound-related pain and patients’ quality of life^(27)^, and pain management techniques^(26,28,35,36,37,39)^.

#### *Subcategory 3* – **Technologies and therapeutic approaches for the relief of physical pain.**

Subcategory 3 encompasses specific treatments, innovative hard technologies, and therapeutic approaches directly aimed at managing pain in complex wounds. Among the examples, virtual reality (VR) and eye tracking stood out, both effective in reducing pain and blood pressure levels. Diode laser treatment has also been described as promising in accelerating the healing of chronic wounds and effectively relieving wound pain, in addition to Transcutaneous Electrical Nerve Stimulation (TENS) therapy and the use of microcurrents. Finally, the use of tissues derived from the amniotic membrane (AM) and umbilical cord (UC) was identified as an effective strategy for the rapid reduction of pain and promotion of closure of chronic and painful ischemic wounds^(8,11,30,33,34,35)^.

#### *Subcategory 4* – **Pharmacological treatments and adjuvant technologies in promoting pain relief.**

Subcategory 4 addresses pharmacological therapies that are associated with other adjuvant technologies, which together act in promoting physical care. In this context, the classes of anesthetics and analgesics used in pain control stand out, including non-steroidal anti-inflammatory drugs (NSAIDs), such as ibuprofen, which are effective in relieving pain in acute and chronic skin wounds, especially when presented in foam dressings^(22,24,26,29,37,39)^. Furthermore, the use of opioids, such as the fentanyl extended-release patch, in treatments for severe pain has been raised^(38)^. The use of cannabinoids has shown potential to modulate pain-related behavior associated with wounds(21,40).

### Category 2: Elements of the psycho-spiritual context of comfort: Therapeutic approaches for psycho-spiritual coping.

In category 2, the psycho-spiritual dimension of Comfort Theory stood out, whose content included interventions for the person’s physical and spiritual care through complementary or alternative therapeutic approaches in pain management. In this category, acupuncture, cognitive-behavioral therapy, meditation, progressive muscle relaxation, music therapy, and biofeedback stood out as relaxation techniques and effective therapies^(11,13,20)^. These interventions represent the use of unconventional or complementary techniques as valuable resources in pain management, aligning with the principles of promoting comfort and coping with the phenomena intrinsic to living with wounds and pain.

## DISCUSSION

The results presented in this scoping review mapped interventions currently used for pain management in people with complex wounds and provide a comprehensive overview of the strategies adopted in clinical practice.

The publications presented different clinical interventions with an impact on reducing pain associated with complex wounds and, therefore, on promoting relief and comfort for those affected. These effects encourage the continuation of treatment by meeting physical needs, in accordance with the principles of Comfort Theory^(10)^. In this context, the search for interventions that promote not only the relief of physical pain, but also emotional tranquility and transcendence in the face of suffering, is highlighted, to amplify the therapeutic effects of wound care itself.

The use of quality measurement instruments for pain assessment, such as the VAS, has proven essential for measuring pain perception in a standardized and sensitive way in patients with complex wounds. Studies using the VAS have demonstrated its usefulness in identifying pain, contributing to the systematization of personalized care, and greater effectiveness in reducing pain intensity, especially in groups undergoing specific pharmacological interventions, such as the use of ibuprofen-impregnated dressings^(21,22,25,26,27,28,29,30,31,32,35,36,40)^.

Among the interventions analyzed, studies using ibuprofen-impregnated foam dressings as an effective alternative for treating painful wounds stand out, demonstrating clinically significant pain relief and contributing to patient comfort during the healing process^(24,25,26,29,37,39)^. The use of these dressings is a way to address the physical dimension of comfort, a less invasive and potentially safer approach than conventional systemic methods.

In addition to topical agents, the effectiveness of using gauze compresses containing hyaluronic acid has been observed. The results indicated positive effects in reducing pain, decreasing wound size, and improving users’ quality of life, while also showing a good safety profile^(27)^. These findings are corroborated by a multicenter controlled study^(42)^ which evaluated the use of the same intervention in leg ulcers of venous or mixed etiology, and observed a significant reduction in wound area, pain intensity, and a high rate of complete healing.

Recent literature highlights the use of emerging digital technologies, such as virtual reality (VR), as a complementary strategy to conventional analgesic practices in the management of physical pain. Evidence suggests that VR provides immediate relief and, in some cases, lasting effects even after the immersion has ended. Furthermore, maintaining a heart rate below 100 beats per minute during use reinforces its safety^(8,11)^. These findings suggest that immersive and interactive technologies are tools with the potential to promote comfort in environmental and psycho-spiritual dimensions, by acting on cognitive distraction, reducing anxiety, and managing pain.

Alongside digital interventions, light-based therapies, such as low-level laser therapy, are gaining ground in pain management practices. This technology uses concentrated light beams, in the case of a diode laser, with a wavelength of 810 nm to stimulate physiological healing processes and promote analgesic, anti-inflammatory, and reparative effects. The study conducted in the United Kingdom demonstrated that this type of treatment can not only accelerate healing but also effectively relieve associated pain^(34,43)^. Although the intervention shows promise in promoting comfort in pain management for complex wounds, effective implementation in the Brazilian context requires investment in training, infrastructure, local protocols, and research on efficacy and cost.

Furthermore, the application of transcutaneous electrical nerve stimulation and microcurrent therapy stands out as interventions with beneficial effects in both pain modulation and the healing of difficult-to-manage wounds. Accel-Heal Solo therapy, based on pulsed electrical stimulation applied through electrodes positioned away from the wound, has proven effective in controlling pain and edema and reducing the need for local or systemic analgesic medication^(33)^. Similarly, combining microcurrent with standard treatment resulted in a significant reduction in wound size and pain levels in all participants, including discontinuation of tramadol use and improved sleep quality^(32)^.

Although there is still a limited number of studies on the application of biological tissues, such as AM, this approach has shown potential in accelerating the healing of chronic wounds and promoting therapeutic comfort. This effect can be attributed to the natural characteristics of the avascular membrane, which is composed of collagen, natural extracellular matrix, and stem cells^(35)^. These findings reinforce the potential clinical value of these biomaterials as an adjuvant alternative, which is still largely unexplored.

In addition to the non-pharmacological strategies explored, pharmacological resources such as NSAIDs, opioids, and local anesthetics are an essential approach to pain relief, especially in cases of moderate to severe intensity; these can be administered via different routes, depending on the nature and characteristics of the pain. The appropriate choice of drug class and route of administration directly influences therapeutic efficacy and the healing process experience^(38,41)^.

This reinforces the idea that rational and individualized medication management is crucial in controlling pain from complex wounds. In this context, the existence of evidencebased protocols can guide therapeutic decisions in nursing care and inform more effective care plans that are driven by selfperceived comfort needs.

Besides systemic formulations, topical interventions with analgesics have proven effective in promoting comfort and pain relief in chronic wounds. Morphine and diamorphine gels, for example, have been shown to provide relief for up to 24 hours, helping to maintain comfort between dressing changes^(22,44)^. Similarly, the use of 2.75% lidocaine, combined with zinc oxide, has proven to be a fast and long-lasting alternative for pain control, with effects extending for up to four hours, being especially useful in outpatient palliative care settings^(45)^. These findings emphasize the value of topical drug application as an adjunctive strategy in wound pain management, with a direct impact on patients’ comfort experience.

Another relevant approach was the use of transdermal fentanyl patches, which allowed for the continuous and controlled release of the opioid, maintaining stable analgesic levels in the blood and reducing the occurrence of side effects^(38)^. Additionally, alternative therapies such as topical medicinal cannabis were also identified. Case studies and recent evidence indicate that modulation of the endocannabinoid system, through the topical application of cannabis oil and the use of opioids, presents analgesic potential in chronic wounds and pyoderma gangrenosum, with pain reduction^(21,40)^.

Still within the physical context of comfort, phytotherapeutic interventions have proven effective. Evidence suggests that Horse Chestnut Seed Extract (HCSE) promoted a statistically significant reduction in leg pain compared to placebo. This effect is attributed to the anti-inflammatory and venotonic properties of the extract, which help reduce edema, improve blood circulation, and promote healing^(28)^. Therefore, HCSE was considered a complementary alternative for pain relief and promotion of physical comfort in people with venous ulcers and other chronic wounds.

After exploring strategies aimed at relieving physical pain, it is necessary to broaden the perspective to other equally relevant dimensions in the care process. In this context, psycho-spiritual comfort^(10)^ involves coping with negative emotions, anxiety, hopelessness, and rediscovering the meaning of life, aspects that directly influence recovery and quality of life. Interventions targeting this area contribute to promoting the patient’s overall well-being.

In this dimension, the acupuncture technique “*Surrounding the Dragon*” (SD) is highlighted, whose effectiveness has been demonstrated in the treatment of various dermatological conditions, such as pressure ulcers, herpes zoster, non-segmental vitiligo, and skin aging^(19)^. This approach aligns with integrative care practices, aiming to promote not only physical benefits, but also relaxation, a sense of well-being, and energy balance—phenomena that influence individuals’ pain experiences.

Other strategies widely used in pain management and psycho-spiritual comfort promotion include cognitive-behavioral therapy, mindfulness, meditation, progressive muscle relaxation, and biofeedback^(11)^. These techniques have a recognized effect on reducing anxiety, regulating emotions, and reframing painful experiences, aspects directly related to the psycho-spiritual domain of the theory.

The interventions identified include technologies such as virtual reality, laser therapy, and medicinal cannabis, which stand out as innovative strategies potentially applicable to the Brazilian context, especially in outpatient services^(8,46,47,48)^. These are viable technologies that are expanding in the country, aligned with contemporary care trends and capable of improving pain management in people with complex wounds.

Among the limitations of this review, it was difficult to find interventions encompassing all domains of Kolcaba’s Theory of Comfort, with a predominance of approaches focused on physical and psycho-spiritual contexts. This limitation can be attributed to the restricted explicit use of theory as a framework in studies, the limitations in operationalizing the sociocultural and environmental domains in clinical practice, and the tendency in the literature to prioritize more objective and easily measurable interventions.

Likewise, there is a predominance of international studies and low levels of evidence in part of the sample, which limits its direct application to the Brazilian reality. However, this international perspective can represent an opportunity to identify and adapt successful strategies and contribute to improving care in the national context, provided that local particularities are respected.

## CONCLUSION

The study allowed the mapping of interventions currently used in pain management in people with complex wounds and indicates that, above all, physical and pharmacological interventions have been implemented and contribute to the immediate experience of relief and tranquility, as proposed by Kolcaba. It should be noted that they also promoted an improvement in quality of life, favoring the reduction in wound size and optimizing the healing process.

However, given the high prevalence of pain associated with complex wounds, the scarcity of interventions, especially in the Brazilian context, that specifically address pain management in this population is striking. Therefore, the study provides theoretical support with the potential to equip person-centered professional care, sensitive to the physical and psycho-spiritual dimensions of Kolcaba’s Comfort Theory. In a clinical context, the findings contribute to pointing out possibilities for more assertive and humanized nursing decisions, strengthening the implementation of evidence-based practices that, through pain relief, also promote overall well-being.

Thus, the research aligns with the principles of Nursing as a science of care and responds to contemporary demands for scientific production that support more effective and humanistic interventions in the care of the person, and not just the treatment of highly complex wounds. In this regard, the need for the development of national studies guiding the development of evidence-based care protocols grounded in consistent theoretical frameworks is highlighted.

## Data Availability

The entire dataset supporting the results of this study was published in the article itself.
