# Supplementary material for: Pain management in complex wounds: a scoping review and analysis using comfort theory
Source: Rev Esc Enferm USP. 2026 Feb 27;60:e20250439. doi: 10.1590/1980-220X-REEUSP-2025-0439en (PMC12955372; doi:10.1590/1980-220X-REEUSP-2025-0439en)
Supplement: Translation

# Translation: Manejo da dor em feridas complexas: revisão de escopo e análise pela teoria do Conforto

**DOI:** 10.1590/1980-220X-REEUSP-2025-0439pt

**Authors:** Mariana Érica da Silva Paixão, Yzis Oliveira Pontes Pereira, Amanda Meireles Medeiros, Rute Xavier Silva, Vívian Lopes Miele, Jocelly de Araújo Ferreira, Oriana Deyze Correia Paiva Leadebal

**Affiliations:** 1 Universidade Federal da Paraíba, Departamento de Enfermagem Clínica, João Pessoa, PB, Brasil.

**Keywords:** Manejo da Dor, Ferimentos e Lesões, Dor Crônica, Protocolos Clínicos, Analgesia

## Abstract

**Objetivo:**

Mapear, sob o olhar da Teoria do Conforto de Kolcaba, evidências científicas de intervenções para manejo de dor em pessoas com feridas complexas.

**Método:**

Revisão de escopo conduzida em sete bases de dados e na literatura cinzenta. Utilizou-se o aplicativo *Rayyan* para seleção do material. O processamento dos dados ocorreu por meio do software IRaMuTeQ, utilizando Classificação Hierárquica Descendente, a partir do tópico resultados da amostra.

**Resultados:**

A amostra, constituída por 27 publicações, compôs o *corpus* textual. As intervenções para o manejo da dor em pessoas com feridas complexas contemplaram dimensões físicas e psicoespirituais da Teoria do Conforto, com prevalência para curativos impregnados com ibuprofeno, tecnologias como realidade virtual, rastreamento ocular e laser de diodo, além de farmacoterapias e abordagens complementares, a exemplo das técnicas de relaxamento, musicoterapia e terapia-cognitivo comportamental.

**Conclusão:**

Essa revisão mapeou intervenções no manejo da dor em feridas complexas, evidenciou benefícios como alívio imediato, melhora na qualidade de vida e favorecimento da cicatrização, em consonância com a Teoria do Conforto de Kolcaba.

## INTRODUÇÃO

Feridas complexas são definidas como toda perda de continuidade da estrutura anatômica da pele ou tecidos mais profundos, não apresentam progressão adequada no processo de cicatrização e, como tal, prolongam-se por seis semanas ou mais, apesar de intervenções terapêuticas ativas. Nestas feridas, ocorrem mecanismos ineficazes de reparação tecidual, configurando desafio para o manejo clínico. Sabe-se que algumas condições comórbidas, como vasculopatias, neuropatias, neoplasias, imobilidade física e déficits nutricionais, corroboram a cronificação de lesões^(1,2,3)^.

O prolongamento do tempo de cicatrização e a presença de comorbidades que comprometem a regeneração celular contribuem para o agravamento da complexidade dessas feridas. Como consequência, há impactos na esfera física, emocional e social das pessoas acometidas, com comprometimento da qualidade de vida. Além disso, são requeridos tratamentos prolongados e contínuos, geradores de custos expressivos aos sistemas de saúde^(2,4)^.

A prevalência de feridas complexas na população mundial ainda não é totalmente conhecida por múltiplos fatores que corroboram a ocorrência dessa comorbidade, porém, estima-se que 20 milhões de pessoas são portadoras destas lesões globalmente, dentre os quais 5 milhões são brasileiros^(5)^.

Do ponto de vista fisiopatológico, sabe-se que a ocorrência de feridas complexas está relacionada frequentemente a condições crônicas de base e alterações na perfusão tecidual. A literatura atual aponta, como tipos mais prevalentes, lesões por pressão (LPP), úlceras vasculares (arteriais e venosas), úlceras diabéticas e feridas cirúrgicas que cicatrizam por segunda intenção. Tais etiologias, embora distintas em sua origem, geram desafios clínicos para a promoção da cicatrização em contextos de comprometimento sistêmico e local, exigindo abordagens terapêuticas individualizadas e multiprofissionais, com evidente protagonismo da Enfermagem^(6,7)^.

Outrossim, o cuidado clínico às pessoas com feridas complexas envolve a gestão de diversas necessidades associadas à convivência com a ferida, entre elas o manejo da dor, um fenômeno multifatorial, que abrange dimensões sensoriais, emocionais e sociais, o que torna o manejo desafiador^(6)^.

A origem das feridas complexas não se limita ao processo fisiopatológico da ferida, estando relacionada a comorbidades como doenças vasculares, Diabetes Mellitus, Hipertensão Arterial Sistêmica e neuropatias, que intensificam as sensações álgicas. Estes estímulos dolorosos são agravados durante o processo de limpeza, devido à alta sensibilidade da área afetada e às intervenções mecânicas envolvidas, como remoção da cobertura primária, higienização, desbridamento de tecidos inviáveis, e aplicação de nova cobertura. Tais procedimentos, embora essenciais ao tratamento, são frequentemente descritos como momentos de experiências álgicas importantes^(6,8)^.

Ao considerar o contexto multifatorial e subjetivo, destaca-se que a meta do cuidado de enfermagem para o manejo clínico da dor nem sempre será a analgesia, dada a natureza crônica de feridas, podendo minimamente constituir promoção de conforto, definido por Katharine Kolcaba como uma experiência imediata de fortalecimento a partir das necessidades de alívio, tranquilidade ou transcendência, que são atendidas nos contextos físicos (referindo-se às sensações corpóreas), psicoespirituais (significado da vida/espiritualidade, o que inclui autoestima, autoconceito), ambientais (relacionados aos elementos do ambiente) e socioculturais (envolve relações interpessoais, familiares, sociais e financeiros)^(9,10)^.

A literatura reúne revisões que descrevem intervenções farmacológicas e não farmacológicas para o manejo da dor no contexto de feridas complexas, com avanços em tecnologias, estratégias analgésicas e abordagens complementares, como técnicas de relaxamento, musicoterapia e terapia cognitivo-comportamental^(11,12,13,14)^. Contudo, tais produções, embora relevantes, apresentam foco predominantemente clínico e biomédico, sem organizar o cuidado em uma perspectiva ampliada que contemple as dimensões de conforto do paciente.

Observa-se, portanto, uma lacuna de pesquisa que justifica o desenvolvimento e a sistematização de intervenções fundamentadas em referenciais teóricos de enfermagem, em especial na Teoria do Conforto de Kolcaba. Esse referencial, por compreender o conforto como resultado mensurável do cuidado e como instrumento que orienta o processo de trabalho da Enfermagem, permite uma abordagem sistematizada, holística e centrada no paciente.

A partir destas considerações, o presente estudo objetivou mapear as evidências científicas de intervenções para manejo de dor, em pessoas com feridas complexas, sob o olhar da teoria do conforto de Kolcaba.

## MÉTODO

### Tipo de Estudo

Foi desenvolvido estudo de revisão, do tipo escopo, conduzido de acordo com as diretrizes metodológicas de JBI para revisão de escopo e conforme as recomendações da diretriz *Preferred Reporting Items for Systematic Reviews and Meta-Analyses extension for Scoping Reviews* (PRISMA-ScR)^(15,16)^. O protocolo de revisão foi registrado no *Open Science Framework* (OSF) sob o DOI 10.17605/OSF.IO/Q3B25 para deixar públicas as informações do estudo, estimular a transparência e possibilitar que a estratégia de busca da pesquisa seja replicada.

A revisão foi desenvolvida nas seguintes etapas: identificação da questão da pesquisa, identificação de estudos relevantes, seleção do estudo, mapeamento dos dados, coleta, resumo e relato dos resultados^(17)^.

### Questão da Pesquisa

Foi utilizada a estratégia mnemônica População, Conceito e Contexto (PCC), sendo População – Adultos com feridas complexas; Conceito – intervenções realizadas para manejo da dor; e Contexto – serviço de atenção à saúde, que orientou a elaboração da questão norteadora da revisão: “Quais são as evidências científicas disponíveis na literatura sobre intervenções de manejo de dor em pessoas com feridas complexas, desenvolvidas em serviços de saúde?” A busca foi realizada de dezembro de 2024 a abril de 2025.

### Critérios de Elegibilidade

Para a seleção do material que compôs o corpus textual, foram adotados como critérios de inclusão: artigos originais completos, disponibilizados na íntegra e gratuitamente em meio eletrônico (acesso CAFe); dissertações e teses, sem restrição de idiomas e sem recorte temporal. Constituíram critérios de exclusão: cartas ao editor e carta ao leitor, resumos em anais de eventos, artigos incompletos, estudos em fase de projeto ou ainda sem resultados.

### Estratégia de Busca

Foi realizada busca nas bases *National Library of Medicine and National Institutes of health* (PUBMED), *Web of Science* (WOS), *Excerpta Medica dataBASE* (Embase), *Cumulative Index to Nursing and Allied Health Literature* (CINAHL), SCOPUS (Elsevier), *Cochrane Library*, Literatura Latino-americana e do Caribe em Ciências da Saúde (LILACS), acessadas através da Comunidade Acadêmica Federada (CAFe), utilizando-se termos constantes no *Medical Subject Headings* (MeSH) e Descritores em Ciências da Saúde (DeCS). A revisão também abrangeu a literatura não convencional (literatura cinzenta) do repositório *ProQuest Dissertations and Thesis Global*, Biblioteca Digital Brasileira de Teses e Dissertações (BDTD) e Google Scholar (limitado aos 100 primeiros resultados).

Os termos-chave utilizados foram ‘Manejo da Dor’, ‘Ferimentos e Lesões’, ‘Dor Crônica’, ‘Protocolos Clínicos’ e ‘Analgesia’. Para aumentar a sensibilidade da pesquisa, esses termos foram combinados com suas traduções correspondentes, como ‘*Pain Management’*, ‘*Wounds and Injuries’*, ‘*Chronic Pain*’, ‘*Clinical Protocols*’ e ‘*Analgesia*’; utilizaram-se os operadores booleanos OR e AND. Uma estratégia de busca específica foi desenvolvida para cada base de dados, com respeito às suas particularidades, com objetivo de otimizar a recuperação de informações relevantes conforme descrito no Quadro 1.

**Quadro 1:** Estratégias de busca utilizadas nas bases de dados – João Pessoa, PB, Brasil, 2025.

Base de dados	Estratégia de busca
MEDLINE/PubMed	(“Pain Management”[MeSH Terms] OR “Pain Management”[Title/Abstract]) AND (“Wounds and Injuries”[MeSH Terms] OR “Wounds and Injuries”[Title/Abstract]) AND (“Chronic Pain”[MeSH Terms] OR “Chronic Pain”[Title/Abstract])
Embase	(‘complex wounds’ OR ‘chronic wounds’:ti,ab,kw OR ‘chronic wounds’) AND (‘pain management’:ti,ab,kw OR ‘pain management’) AND (‘analgesia’:ti,ab,kw OR ‘analgesia’)
SCOPUS	(TITLE-ABS-KEY (“Complex Wounds”) OR TITLE-ABS-KEY (“Wounds and Injuries”) AND TITLE-ABS-KEY (“Pain Management”) AND TITLE-ABS-KEY (“Chronic Pain”))
*Web of Science*	TS = (“Pain management”) AND TS = (“Wounds and injuries”) OR TS = (“Complex Wounds”) OR TS = (“Chronic Wounds”) AND TS = (“Chronic Pain”) AND TS = (“Clinical Protocols”) OR TS = (“Treatment Protocols”)
LILACS	(“Manejo da Dor”) OR (“Manejo del Dolor”) AND (“Ferida”) OR (“Herida”) OR (“Feridas”) OR (“Heridas”) AND (“Dor Crônica”) OR (“Dolor Crónico”) AND (“Cuidados de Enfermagem”) OR (“Atención de Enfermería”) OR (“Assistência de Enfermagem”) OR (“Cuidado de Enfermería”) AND (instance:”regional”)
*Cochrane Library*	“Pain Management” in Title Abstract Keyword AND “Wounds and Injuries” in Title Abstract Keyword OR “Complex Wounds” in Title Abstract Keyword OR Injury in Title Abstract Keyword AND “Chronic Pain” in Title Abstract Keyword
CINAHL	“Pain Management” AND (“Wounds and Injuries”) OR Injury AND “Chronic Pain” AND “Clinical Protocols”
Biblioteca Digital Brasileira de Teses e Dissertações	Feridas AND “Manejo da Dor”
ProQuest	(“Complex Wounds” OR “Chronic Wounds”) AND “Chronic Pain”
Google Scholar	(“Complex Wounds” OR “Chronic Wounds” OR “Wounds and Injuries” OR Injury) AND “Pain Management” AND “Chronic Pain” OR “Analgesia”

Fonte: Dados da pesquisa, 2025.

### Seleção dos Estudos

Após a pesquisa nas bases de dados, todos os registros encontrados foram exportados para o gerenciador de referências Zotero, versão 7.0.10, para exclusão de duplicatas. A seleção e avaliação dos estudos foi realizada por dois revisores de forma independente e às cegas, recorrendo-se a um terceiro revisor nos casos de divergências, por meio do aplicativo da *web Rayyan*. A pré-seleção dos estudos foi realizada mediante leitura do título e resumos, baseando-se nos critérios de inclusão e exclusão. Os estudos pré-selecionados foram então submetidos à leitura na íntegra para a definição dos elegíveis.

Por fim, foi realizada uma busca nas listas de referências dos estudos incluídos, a fim de que fossem identificados outros artigos não localizados em buscas anteriores. Os estudos considerados elegíveis nesta etapa foram incluídos aos demais previamente selecionados, e fizeram parte do *corpus da scoping review*. O processo de seleção é apresentado, conforme o Diagrama de fluxo do PRISMA 2020.

### Extração dos Dados

Para a extração das informações do *corpus* foi utilizado um instrumento elaborado pelos pesquisadores, estruturado em uma planilha no Microsoft Excel, com as seguintes variáveis: base, periódico, ano de publicação, autoria, idioma, DOI, título, país de origem, objetivo, delineamento metodológico, principais resultados e conclusão.

### Apresentação dos Dados

Elaborou-se uma síntese dos conteúdos de cada estudo, dando origem ao corpus textual, que por sua vez foi submetido ao *software Interface de R pour les Analyses Multidimensionnelles de Textes et de Questionnaires* (IRaMuTeQ) versão 0.7 alpha2, por meio da análise multivariada de Classificação Hierárquica Descendente (CHD), que possibilitou o agrupamento de vocabulários semelhantes em classes de segmentos textuais e apresentação das relações semânticas por meio de dendrograma gerado pelo software^(18)^.

Posteriormente à apresentação da CHD proposta pelo software procedeu-se à reorganização das classes (5 classes) segundo análise de conteúdo fundamentada por referencial teórico de Katharine Kolcaba^(10)^, o que permitiu a identificação de 2 categorias.

### Aspectos Éticos

Esta revisão de escopo utilizou exclusivamente dados secundários disponíveis publicamente e, por isso, não demandou aprovação por Comitê de Ética em Pesquisa. Todas as etapas foram conduzidas em conformidade com os princípios éticos e as diretrizes metodológicas cabíveis, assegurando o respeito aos direitos autorais das produções analisadas.

## RESULTADOS

O corpus textual do estudo resultou de 27 artigos dentre os 1.577 encontrados nas bases de dados e repositórios, cujo processo de identificação, triagem e elegibilidade ocorreu conforme Figura 1.

**Figura 1:** Diagrama de fluxo do PRISMA de seleção de estudos e processo de inclusão na revisão de escopo. João Pessoa, PB, Brasil, 2025.

A fim de facilitar a apresentação, optou-se por organizar os dados mais relevantes de caracterização dos artigos em um quadro (Quadro 2).

**Quadro 2:** Caracterização dos estudos – João Pessoa, PB, Brasil, 2025.

ID^*^	Autor/Ano	Título	Objetivo
A1	Bechert e Abraham, 2009^(13)^	Pain management and wound care	Discutir a etiologia da dor, bem como fornecer ao leitor estratégias para gerenciar a ferida dolorosa uma vez que tenha sido identificado.
A2	Ffrench et al., 2023^(19)^	Systematic review of topical interventions for the management of pain in chronic wounds	Estabelecer uma compreensão robusta do nível de eficácia das intervenções tópicas disponíveis para o tratamento da dor associada a feridas em indivíduos com experiência vivida de feridas crônicas.
A3	Guglielmo, 2024^(20)^	The ‘Surrounding the Dragon’ (Wei Ci) Acupuncture Technique: A Systematic Review	Determinar a eficácia baseada em evidências da antiga técnica de acupuntura da medicina tradicional chinesa (MTC) ‘Surrounding the dragon’.
A4	Healy et al., 2023^(21)^	Chronic wound-related pain, wound healing and the therapeutic potential of cannabinoids and endocannabinoid system modulation	Resumir de forma abrangente as descobertas até o momento sobre o potencial dos canabinoides, moduladores do sistema endocanabinoide e medicamentos à base de cannabis para o tratamento de dor crônica relacionada a feridas e sua cicatrização.
A5	Mervis e Federman, 2018^(11)^	Pain Management in Patients with Chronic Wounds	Fornecer uma estrutura baseada em evidências para o gerenciamento farmacológico e não farmacológico da dor em pacientes com feridas crônicas.
A6	Metcalfe, 2015^(22)^	Pain management in palliative patients with chronic wounds	Definir a dor em relação a feridas paliativas crônicas, examinar as incidências e taxas de dor crônica neste grupo de pacientes e criticar a literatura para as melhores estratégias de gerenciamento.
A7	Price et al., 2007^(12)^	Managing painful chronic wounds: the Wound Pain Management Model	Avaliar a dor da ferida e estratégias de tratamento, com foco na dor crônica persistente e na dor associada ao tratamento local da ferida, incluindo troca de curativo.
A8	Richardson, 2012^(23)^	An introduction to the biopsychosocial complexities of managing wound pain	Discutir o tratamento de feridas agudas e crônicas, com foco nas habilidades específicas necessárias para gerenciar a dor associada a diferentes tipos de feridas e abordar as áreas importante de troca de curativos.
A9	Woo et al., 2013^(24)^	Evidence-based approach to manage persistent wound-related pain	Fornecer uma abordagem baseada em evidências para o tratamento da dor persistente em pessoas com feridas crônicas.
A10	Arapoglou et al., 2011^(25)^	Analgesic efficacy of ibuprofen-releasing foam dressing compared with local best practice for painful exuding wounds	Examinar se a etiologia da ferida tem efeito nas propriedades analgésicas de um curativo de espuma liberadora de ibuprofeno, que anteriormente demonstrou reduzir a dor em feridas de várias etiologias, em comparação com as melhores práticas locais (LBP).
A11	Araújo et al., 2021^(8)^	Realidade virtual durante o manejo da dor em pessoas com feridas crônicas: estudo experimental	Avaliar o uso de realidade virtual em ambientes de imersão e simulações realísticas para o alivio da dor em pacientes com feridas crônicas durante a troca do curativo.
A12	Gottrup et al., 2007^(26)^	Less pain with Biatain–Ibu: initial findings from a randomised, controlled, double-blind clinical investigation on painful venous leg ulcers	Investigar se o curativo de espuma de ibuprofeno alivia a dor da úlcera venosa sem comprometer as propriedades benéficas da cicatrização úmida da ferida, com um perfil de segurança aceitável.
A13	Humbert et al., 2013^(27)^	Efficacy and safety of a gauze pad containing hyaluronic acid in treatment of leg ulcers of venous or mixed origin: a double-blind, randomised, controlled trial	Investigar a eficácia e segurança de uma compressa de gaze contendo ácido hialurônico (AH) no tratamento local de úlceras venosas nas pernas, em comparação com seu veículo neutro.
A14	Pittler e Ernst, 2012^(28)^	Horse chestnut seed extract for chronic venous insufficiency	Revisar a eficácia e a segurança do extrato oral de semente de castanha da Índia (HCSE) versus placebo ou terapia de referência para o tratamento de IVC.
A15	Romanelli et al., 2009^(29)^	Ibuprofen slow-release foam dressing reduces wound pain in painful exuding wounds: preliminary findings from an international real-life study	Comparar o efeito de um curativo de espuma que libera ibuprofeno (Biatain Ibu ®) com as melhores práticas locais no tratamento de feridas exsudativas dolorosas.
A16	Kitala et al., 2022^(30)^	Eye-tracked computer games as a method for pain perception alleviation in chronic wound management	Avaliar a usabilidade de jogos de rastreamento ocular que distraem o paciente durante atividades de tratamento de feridas em uma clínica cirúrgica ambulatorial e, portanto, verificar se vale a pena introduzir rastreadores oculares na rotina clínica diária.
A17	Rook et al., 2019^(31)^	Mepilex® Border Flex — results of an observational study in German specialist wound care centres	Avaliar o tratamento de uma variedade de tipos de feridas exsudativas com um curativo de espuma revestido de silicone autoadesivo.
A18	Nair, 2018^(32)^	Microcurrent as an adjunct therapy to accelerate chronic wound healing and reduce patient pain	Avaliar a eficácia da microcorrente como terapia adjuvante na redução do tamanho da ferida e da pontuação de dor em pacientes com feridas crônicas.
A19	Swift et al., 2024^(33)^	Taking the pain out of wound healing with microcurrent electrical stimulation therapy	Avaliar o uso do Accel-Heal Solo para reduzir dor associada a feridas difíceis de cicatrizar e seu impacto na dependência de analgésicos.
A20	Tang et al.,2023^(34)^	The associations between diode laser (810 nm) therapy and chronic wound healing and pain relief: Light into the chronic wound patient’s life	Determinar as associações entre a terapia com laser de diodo e a cura da ferida crônica ou alívio da dor.
A21	Acevedo, 2020^(35)^	Successful treatment of painful chronic wounds with amniotic and umbilical cord tissue: A case series	Não apresenta.
A22	Bradbury et al., 2008^(36)^	Measuring outcomes with complex patients: an audit of the effect of Actiform Cool on painful wounds	Explorar o efeito do Actiform Cool na dor relacionada à ferida.
A23	Flanagan et al., 2006^(37)^	Case series investigating the experience of pain in patients with chronic venous leg ulcers treated with a foam dressing releasing ibuprofen	Traçar o perfil e as características dos indivíduos utilizando o curativo de espuma com ibuprofeno.
A24	Freedman et al., 2004^(38)^	Practical treatment of pain in patients with chronic wounds: pathogenesis-guided management	Construir uma proposta geral de gerenciamento da dor com base na correção de causas patológicas da ferida crônica.
A25	Hampton, 2007^(39)^	Chronic pain in wounds: a report on 11 case studies	Investigar a eficácia da redução da dor e o impacto sobre a qualidade de vida do curativo impregnado com ibuprofeno.
A26	Maida e Corban, 2017^(40)^	Topical Medical Cannabis: A New Treatment for Wound Pain—Three Cases of Pyoderma Gangrenosum	Avaliar a eficácia e segurança do tratamento com cannabis medicinal tópica composta em óleo de girassol orgânico não geneticamente modificado em pacientes com pioderma gangrenoso.
A27	Eriksson et al., 2021^(41)^	Chronic wounds: Treatment consensus	Formular um consenso imparcial sobre o melhor tratamento de feridas crônicas.

Fonte: Elaborado pelos autores, 2025.

*A: artigo, seguido no número sequencial.

Conforme observa-se no Quadro 2, os estudos foram publicados entre 2004 e 2024, maior ocorrência de publicação nos anos de 2023, com três estudos (11,2%), e em 2024, com dois estudos (7,40%). Frequência semelhante de dois estudos (7,40%) também foi observada nos anos de 2020, 2018, 2013, 2012, 2009, 2008 e 2007, enquanto nos anos de 2022, 2021, 2019, 2017, 2015, 2011, 2006 e 2004 apresentaram apenas um estudo cada (3,70%).

Em relação aos países de origem, destacaram-se o Reino Unido com seis (22,3%), seguido por cinco (18,5%) dos Estados Unidos da América. Canadá, China, Dinamarca e Irlanda contribuíram com dois estudos cada (7,40%). Brasil, Malásia, Itália, Grécia, França, Polônia, Alemanha e Inglaterra contribuíram com um estudo cada (3,70%).

Quanto aos níveis de evidência, nove (33,3%) eram nível I, seis (22,2%) eram de nível II, dois (7,40%) do nível III, três (11,1%) do nível V, seis (22,2%) do nível VI e por fim, um (3,70%) do nível VII. No que diz respeito ao idioma, todos os artigos eram em língua inglesa.

A Figura 2 ilustra o dendrograma proveniente da análise de CHD, composta por 5 classes denominadas segundo a expressão de conteúdo, e oriundas do *corpus* textual, constituído por 27 textos, 116 segmentos textuais e 4.072 palavras, cujo índice de aproveitamento foi de 79,31% dos segmentos processados, considerado um bom percentual de aproveitamento.

**Figura 2:** Dendrograma de Classificação Hierárquica Descendente do *Corpus* Textual processado pelo software IRaMuTeQ. João Pessoa, PB, Brasil, 2025.

Após o exercício analítico, subsidiado pelo referencial teórico de Katharine Kolcaba^(10)^, sobre este *corpus* textual categorizado pelo *software* IRaMuTeQ, surgiram 2 categorias e 4 subcategorias. As categorias identificadas representam intervenções físicas e psicoespirituais para o manejo da dor em feridas complexas, que são apresentadas a seguir, na Figura 3.

**Figura 3:** Categorização do Corpus segundo as intervenções para manejo da dor em pessoas com feridas complexas, baseado nos contextos da Teoria do Conforto. João Pessoa, PB, Brasil, 2025.

### Categoria 1: Elementos do contexto físico do conforto: instrumentos de mensuração da dor física e da eficácia das intervenções; tecnologias e abordagens terapêuticas; e tratamentos farmacológicos na promoção do alívio da dor.

Na categoria 1, destacou-se a dimensão física da Teoria do conforto, que recomenda o uso de instrumentos, intervenções e tecnologias que possibilitem mensurar e aliviar a dor física, favorecendo respostas terapêuticas mais eficazes no cuidado a pessoas com feridas complexas.

#### *Subcategoria 1* – Instrumentos de mensuração da dor física e da eficácia das intervenções

A subcategoria 1 demonstra os instrumentos de mensuração e a sua eficácia nas intervenções no manejo da dor, a exemplo do uso de escalas padronizadas, como Escala Visual Analógica (VAS), que possibilita uma avaliação objetiva da dor, antes e após a implementação das intervenções, ao permitir verificar se houve uma redução estatisticamente significativa dos sintomas álgicos^(21,22,25,26,27,28,29,31,32,35,36,40)^.

#### *Subcategoria 2* – Resultado das intervenções para alívio físico como parte do manejo da dor

A subcategoria 2 reflete os resultados das intervenções físicas de cuidado direto da ferida como parte do manejo da dor. Após as ações clínicas, os conteúdos que remeteram aos resultados foram: reduzir, menor, baixo, melhora, qualidade e diminuir. Essas palavras refletem a efetividade das estratégias, como por exemplo o uso de curativos com gaze contendo ácido hialurônico (HA), que demonstrou ter impacto favorável na dor relacionada à ferida e na qualidade de vida dos pacientes^(27)^, e técnicas de controle de dor^(26,28,35,36,37,39)^.

#### *Subcategoria 3* – Tecnologias e abordagens terapêuticas para o alívio da dor física

A subcategoria 3 abrange tratamentos específicos, tecnologias duras inovadoras e abordagens terapêuticas voltadas diretamente para o manejo da dor em feridas complexas. Entre os exemplos, destacou-se a realidade virtual (VR) e o rastreamento ocular, ambos eficazes na redução dos níveis álgicos e dos níveis pressóricos. O tratamento com laser de diodo também foi descrito como promissor na aceleração da cicatrização de feridas crônicas e alívio efetivo da dor na ferida, além da Terapia de Estimulação Elétrica Nervosa Transcutânea (TENS) e o uso de microcorrentes. Por fim, identificou-se a utilização de tecidos derivados da membrana amniótica (AM) e cordão umbilical (UC) como estratégia eficaz para a rápida redução da dor e promoção do fechamento de feridas crônicas e isquêmicas dolorosas^(8,11,30,33,32,33,34,35)^.

#### *Subcategoria 4* – Tratamentos farmacológicos e tecnologias adjuvantes na promoção do alívio da dor

A subcategoria 4 aborda as terapias farmacológicas que estão associadas a outras tecnologias adjuvantes, que, em conjunto, atuam na promoção do cuidado físico. Nesse contexto, destacamse as classes de anestésicos e analgésicos utilizados no controle da dor, incluindo os anti-inflamatórios não esteroides (AINES), como o ibuprofeno, com eficácia no alívio da dor em feridas cutâneas agudas e crônicas, especialmente quando apresentados em curativos de espuma^(22,24,26,29,37,39)^. Além disso, levantou-se o uso de opioides, como o adesivo de liberação prolongada de fentanil, utilizados em tratamentos para dor intensa^^(38)^^. O uso de canabinoides têm demonstrado potencial para modular o comportamento relacionado à dor inerente a feridas^(21,40)^.

### Categoria 2: Elementos do contexto psicoespiritual do conforto: Abordagens terapêuticas para enfrentamento psicoespiritual.

Na categoria 2, destacou-se a dimensão psicoespiritual da Teoria do conforto, cujo conteúdo contemplou intervenções de cuidado físico e espiritual da pessoa por meio de abordagens terapêuticas complementares ou alternativas no manejo da dor. Nesta categoria destacaram-se como técnicas de relaxamento e terapias de eficácia comprovada a acupuntura, a terapia cognitivo-comportamental, a meditação, o relaxamento muscular progressivo, a musicoterapia e o *biofeedback* ^(11,13,20)^. Essas intervenções representam o uso de técnicas não convencionais ou complementares como recursos valiosos no controle da dor, alinhando-se aos princípios da promoção do conforto e do enfrentamento dos fenômenos intrínsecos à convivência com a ferida e a dor.

## DISCUSSÃO

Os resultados apresentados nesta revisão de escopo mapearam intervenções atualmente utilizadas para o manejo de dor em pessoas com feridas complexas e oferece visão abrangente, das estratégias adotadas na prática clínica.

As publicações apresentaram diferentes intervenções clínicas com impacto na redução da dor associada às feridas complexas e, portanto, de promoção de alívio e conforto às pessoas acometidas. Esses efeitos favorecem a continuidade do tratamento, ao atenderem às necessidades físicas, em consonância com os princípios da Teoria do Conforto^(10)^. Nesse contexto, destaca-se a busca por intervenções que promovam não apenas o alívio da dor física, mas também a tranquilidade emocional e a transcendência frente ao sofrimento, de modo a ampliar os efeitos terapêuticos do cuidado com a ferida propriamente dita.

A utilização de instrumentos de mensuração de qualidade na avaliação da dor, como a VAS, mostrou-se essencial para mensurar de forma padronizada e sensível a percepção dolorosa em pacientes com feridas complexas. Estudos realizados com o uso da VAS evidenciaram sua utilidade na identificação da dor, contribuição na sistematização de cuidado personalizado e maior efetividade sobre a redução da intensidade da dor, especialmente em grupos submetidos a intervenções farmacológicas específicas, como o uso de curativos impregnados com ibuprofeno^(21,22,25,26,27,28,29,30,31,32,35,36,40).^

Dentre as intervenções analisadas, destacam-se os estudos que utilizaram curativos de espuma impregnados com ibuprofeno como uma alternativa eficaz para o tratamento de feridas dolorosas, os quais têm demonstrado alívio da dor clinicamente significativo e contribuído para o conforto do paciente durante o processo de cicatrização^(24,25,26,29,37,39)^. O uso desses curativos configura-se como uma forma de atender à dimensão física do conforto, uma abordagem menos invasiva e potencialmente mais segura do que os métodos sistêmicos convencionais.

Além dos agentes tópicos, foi observada a eficácia do uso de compressas de gaze contendo ácido hialurônico. Os resultados indicaram efeitos positivos na redução da dor, diminuição do tamanho da ferida e melhora na qualidade de vida dos usuários, apresentando ainda um bom perfil de segurança^(27)^. Esses achados são corroborados por um estudo multicêntrico controlado^(42)^, que avaliou o uso da mesma intervenção em úlceras de perna de etiologia venosa ou mista, e observou redução significativa da área da ferida, da intensidade da dor e uma taxa elevada de cicatrização completa.

A literatura recente destaca o uso de tecnologias digitais emergentes, como a realidade virtual (VR), como estratégia complementar às práticas analgésicas convencionais no manejo da dor física. Evidências indicam que a VR promove alívio imediato e, em alguns casos, efeitos prolongados mesmo após o término da imersão. Além disso, a manutenção da frequência cardíaca abaixo de 100 batimentos por minuto durante o uso reforça sua segurança^(8,11)^. Esses achados sugerem que as tecnologias imersivas e interativas são ferramentas com potencial para promover conforto nas dimensões ambiental e psicoespiritual, ao atuar sobre a distração cognitiva, redução da ansiedade e manejo da dor.

Paralelamente às intervenções de natureza digital, terapias baseadas em luz, como o laser de baixa intensidade, vêm ganhando espaço nas práticas assistenciais voltadas à dor. Essa tecnologia utiliza feixes de luz concentrada, no caso do laser de diodo, com comprimento de onda de 810 nm para estimular processos fisiológicos de cicatrização, e promover efeitos analgésicos, anti-inflamatórios e reparadores. O estudo realizado no Reino Unido demonstrou que esse tipo de tratamento pode não apenas acelerar a cicatrização mas também aliviar de forma eficaz a dor associada^(34,43)^. Apesar de a intervenção mostrar-se promissora na promoção de conforto no manejo da dor em feridas complexas, a implementação efetiva na realidade brasileira requer investimento em capacitação, infraestrutura, protocolos locais e pesquisas sobre eficácia e custo.

Outrossim, destaca-se a aplicação da estimulação elétrica transcutânea e da microcorrente, como intervenções com efeitos benéficos tanto na modulação da dor quanto na cicatrização de feridas de difícil manejo. A terapia Accel-Heal Solo, baseada em estimulação elétrica pulsada aplicada por eletrodos posicionados distantes da ferida, mostrou-se eficaz no controle da dor e do edema e na diminuição da necessidade de medicamento analgésicos locais ou sistêmicos^(33)^. De maneira semelhante, a combinação da microcorrente com o tratamento padrão resultou em significativa redução do tamanho das feridas e dos níveis de dor em todos os participantes, inclusive com suspensão do uso de tramadol e melhora na qualidade do sono^(32)^.

Embora ainda haja um número limitado de estudos sobre a aplicação de tecidos biológicos, como a AM, essa abordagem tem demonstrado potencial na aceleração da cicatrização de feridas crônicas e na promoção do conforto terapêutico. Esse efeito pode ser atribuído às características naturais da membrana avascular composta por colágeno, matriz extracelular natural e células-tronco^(35)^. Esses achados reforçam o potencial valor clínico desses biomateriais como alternativa adjuvante, ainda pouco explorada.

Adicionalmente às estratégias não farmacológicas exploradas, os recursos farmacológicos como AINEs, opioides e anestésicos locais, compõem abordagem essencial para o alívio da dor, especialmente em casos de intensidades moderadas a intensas; esses podem ser administrados por diferentes vias, conforme natureza e características da dor. A escolha adequada da classe e da via de administração influencia diretamente a eficácia terapêutica e a experiência do processo de cicatrização^(38,41)^.

Isso reforça que a administração racional e individualizada de medicamentos é crucial no controle da dor em feridas complexas. Nesse cenário, a existência de protocolos baseados em evidências pode orientar decisões terapêuticas ao cuidado de enfermagem e instruir planos de cuidado mais eficazes e orientados por necessidades de conforto autopercebidas.

Além das formulações sistêmicas, intervenções tópicas com analgésicos têm se mostrado eficazes na promoção do conforto e alívio da dor em feridas crônicas. Géis de morfina e diamorfina, por exemplo, demonstraram proporcionar alívio por até 24 horas, contribuindo para a manutenção do conforto entre os intervalos entre um curativo e outro^(22,44)^. De forma semelhante, o uso de lidocaína a 2,75%, associada ao óxido de zinco, mostrou-se uma alternativa rápida e duradoura para o controle da dor, com efeitos que se estendem por até quatro horas, sendo especialmente útil em contextos de cuidados paliativos ambulatoriais^(45)^. Esses achados reforçam o valor da aplicação tópica de fármacos como estratégia adjuvante no manejo da dor de feridas, com impacto direto na experiência de conforto dos pacientes.

Outra abordagem relevante foi o uso de adesivo transdérmico de fentanil, que possibilitou a liberação contínua e controlada do opioide, mantendo níveis analgésicos estáveis no sangue e reduzindo a ocorrência de efeitos colaterais^(38)^. De forma complementar, terapias alternativas como a cannabis medicinal tópica também foram identificadas. Estudos de caso e evidências recentes indicam que a modulação do sistema endocanabinoide, por meio da aplicação tópica de óleo de cannabis e do uso de opioides, apresenta potencial analgésico em feridas crônicas e pioderma gangrenoso, com redução da dor^(21,40)^.

Ainda no contexto físico do conforto, intervenções fitoterápicas têm se mostrado eficazes. Evidências apontam que o Extrato de Semente de Castanha da Índia (HCSE) promoveu redução estatisticamente significativa da dor nas pernas em comparação ao placebo. Esse efeito é atribuído às propriedades anti-inflamatórias e venotônicas do extrato, que auxiliam na diminuição do edema, melhoram a circulação sanguínea e favorecem a cicatrização^(28)^. Dessa forma, o HCSE foi considerado como uma alternativa complementar para o alívio da dor e promoção do conforto físico em pessoas com úlceras venosas e outras feridas crônicas.

Após explorar as estratégias voltadas ao alívio da dor física, é necessário ampliar o olhar para outras dimensões igualmente relevantes no processo de cuidado. Neste contexto, o conforto psicoespiritual^(10)^ envolve o enfrentamento de emoções negativas, ansiedade, desesperança e o resgate do sentido da vida, aspectos que influenciam diretamente a recuperação e a qualidade de vida. Intervenções direcionadas a essa esfera contribuem para a promoção do bem-estar global do paciente.

Nesta dimensão, destacou-se a técnica de acupuntura “*Surrounding the Dragon*” (SD), cuja eficácia foi evidenciada no tratamento de diversas condições dermatológicas, como úlcera de pressão, herpes zoster, vitiligo não segmentar e envelhecimento da pele^(19)^. Essa abordagem alinha-se às práticas de cuidado integrativo, para promover não apenas benefícios físicos, mas também relaxamento, sensação de bem-estar e equilíbrio energético, fenômenos intervenientes sobre as experiências álgicas dos indivíduos.

Outras estratégias amplamente empregadas no manejo da dor e na promoção do conforto psicoespiritual foram a terapia cognitivo-comportamental, atenção plena, meditação, relaxamento muscular progressivo e *biofeedback* ^(11)^. Essas técnicas têm seu efeito reconhecido sobre a redução da ansiedade, a regulação emocional e a ressignificação da experiência dolorosa, aspectos diretamente relacionados ao domínio psicoespiritual da teoria.

As intervenções identificadas incluem tecnologias como realidade virtual, laserterapia e cannabis medicinal, as quais despontam como estratégias inovadoras e potencialmente aplicáveis ao contexto brasileiro, sobretudo em serviços ambulatoriais^(8,46,47,48)^. Trata-se de tecnologias viáveis e em expansão no país, alinhadas às tendências contemporâneas de cuidado e capazes de qualificar o manejo da dor em pessoas com feridas complexas.

Dentre as limitações desta revisão, houve dificuldade em identificar intervenções que contemplassem todos os domínios da Teoria do Conforto de Kolcaba, com predomínio das abordagens voltadas aos contextos físico e psicoespiritual. Essa limitação pode ser atribuída à pouca utilização explícita da teoria como referencial nos estudos, às limitações de operacionalização dos domínios sociocultural e ambiental na prática clínica, e à tendência da literatura em priorizar intervenções mais objetivas e facilmente mensuráveis.

Além disso, há predominância de estudos internacionais e níveis de evidência baixos em parte da amostra, o que limita a sua aplicação direta na realidade brasileira. No entanto, essa perspectiva internacional pode representar uma oportunidade para identificação e adaptação de estratégias bem-sucedidas e contribuir para o aprimoramento da assistência no contexto nacional, desde que respeitadas as particularidades locais.

## CONCLUSÃO

O estudo possibilitou o mapeamento de intervenções atualmente utilizadas no manejo da dor em pessoas com feridas complexas e indica que sobretudo as intervenções físicas e farmacológicas têm sido implementadas e contribuem para a experiência imediata de alívio e tranquilidade, conforme proposto por Kolcaba. Destaca-se que elas também promoveram melhora na qualidade de vida, favorecendo a redução do tamanho das feridas e otimizando os processos cicatriciais.

Contudo, dada a alta prevalência de dor associada a feridas complexas, chama atenção a escassez de intervenções, especialmente no contexto brasileiro, que abordem de forma específica o manejo da dor nesse público. Logo, o estudo fornece subsídios teóricos com potencial para instrumentalizar o cuidado profissional centrado na pessoa, sensível às dimensões física e psicoespiritual da Teoria do Conforto de Kolcaba. No contexto clínico, os achados contribuem para apontar possibilidades a decisões de enfermagem mais assertivas e humanizadas, fortalecendo a implementação de práticas baseadas em evidências que, por meio do alívio da dor, também promovam o bem-estar integral.

Assim, a pesquisa alinha-se aos princípios da Enfermagem como ciência do cuidado e responde a demandas contemporâneas de produção científica que subsidiem intervenções mais efetivas e humanísticas do cuidado da pessoa e não apenas o tratamento de feridas de alta complexidade. Neste sentido, ressalta-se a necessidade do desenvolvimento de estudos nacionais que orientem o desenvolvimento de protocolos de cuidado baseado em evidências e fundamentados por referenciais teóricos consistentes.

## Data Availability

Todo o conjunto de dados que dá suporte aos resultados deste estudo foi publicado no próprio artigo.

# Translation: Manejo del dolor en heridas complejas: revisión del alcance y análisis utilizando la teoría del confort

**DOI:** 10.1590/1980-220X-REEUSP-2025-0439es

**Authors:** Mariana Érica da Silva Paixão, Yzis Oliveira Pontes Pereira, Amanda Meireles Medeiros, Rute Xavier Silva, Vívian Lopes Miele, Jocelly de Araújo Ferreira, Oriana Deyze Correia Paiva Leadebal

**Affiliations:** 1 Universidade Federal da Paraíba, Departamento de Enfermagem Clínica, João Pessoa, PB, Brasil.

**Keywords:** Manejo del Dolor, Heridas y Lesiones, Dolor Crónico, Protocolos Clínicos, Analgesia

## Abstract

**Objetivo:**

Mapear, desde la perspectiva de la Teoría del Confort de Kolcaba, la evidencia científica de las intervenciones para el manejo del dolor en personas con heridas complejas.

**Método:**

Revisión del alcance realizada en siete bases de datos y literatura gris. La aplicación *Rayyan* para la selección de materiales fue utilizada. El procesamiento de los datos se realizó mediante el software IRaMuTeQ, empleando la Clasificación Jerárquica Descendente, en función de la temática de los resultados de la muestra.

**Resultados:**

La muestra, compuesta por 27 publicaciones, comprendió el *corpus* textual. Las intervenciones para el manejo del dolor en personas con heridas complejas abarcaron dimensiones físicas y psicoespirituales de la Teoría del Confort, con prevalencia de apósitos impregnados con ibuprofeno, tecnologías como la realidad virtual, el seguimiento ocular y el láser de diodo, además de farmacoterapias y enfoques complementarios, como técnicas de relajación, musicoterapia y terapia cognitivo-conductual.

**Conclusión:**

Esta revisión trazó un mapa de las intervenciones en el manejo del dolor de heridas complejas, destacando beneficios como el alivio inmediato, la mejora de la calidad de vida y la promoción de la curación, de acuerdo con la Teoría del Confort de Kolcaba.

## INTRODUCCIÓN

Las heridas complejas se definen como cualquier pérdida de continuidad de la estructura anatómica de la piel o de los tejidos más profundos, que no presentan una progresión adecuada en el proceso de cicatrización y, como tales, se prolongan durante seis semanas o más, a pesar de las intervenciones terapéuticas activas. En estas heridas se producen mecanismos ineficaces de reparación tisular, lo que supone un reto para el manejo clínico. Se sabe que algunas afecciones comórbidas, como las vasculopatías, las neuropatías, las neoplasias, la inmovilidad física y los déficits nutricionales, contribuyen a la cronificación de las lesiones^(1,2,3)^.

La prolongación del tiempo de cicatrización y la presencia de comorbilidades que comprometen la regeneración celular contribuyen a agravar la complejidad de estas heridas. Como consecuencia, se producen impactos en la esfera física, emocional y social de las personas afectadas, lo que compromete su calidad de vida. Además, se requieren tratamientos prolongados y continuos, que generan costos significativos para los sistemas de salud^(2,4)^.

La prevalencia de heridas complejas en la población mundial aún no se conoce del todo debido a múltiples factores que corroboran la aparición de esta comorbilidad, pero se estima que 20 millones de personas padecen estas lesiones en todo el mundo, de las cuales 5 millones son brasileñas^(5)^.

Desde el punto de vista fisiopatológico, se sabe que la aparición de heridas complejas suele estar relacionada con afecciones crónicas subyacentes y alteraciones en la perfusión tisular. La literatura actual señala como tipos más prevalentes las lesiones por presión (LPP), las úlceras vasculares (arteriales y venosas), las úlceras diabéticas y las heridas quirúrgicas que cicatrizan por segunda intención. Estas etiologías, aunque distintas en su origen, generan retos clínicos para promover la cicatrización en contextos de compromiso sistémico y local, lo que exige enfoques terapéuticos individualizados y multiprofesionales, con un protagonismo evidente de la enfermería^(6,7)^.

Además, la atención clínica a las personas con heridas complejas implica la gestión de diversas necesidades asociadas a la convivencia con la herida, entre ellas el manejo del dolor, un fenómeno multifactorial que abarca dimensiones sensoriales, emocionales y sociales, lo que dificulta su manejo^(6)^.

El origen de las heridas complejas no se limita al proceso fisiopatológico de la herida, sino que está relacionado con comorbilidades como enfermedades vasculares, diabetes mellitus, hipertensión arterial sistémica y neuropatías, que intensifican las sensaciones de dolor. Estos estímulos dolorosos se agravan durante el proceso de limpieza, debido a la alta sensibilidad de la zona afectada y a las intervenciones mecánicas que se realizan, como la retirada de la cubierta primaria, la higienización, el desbridamiento de tejidos inviables y la aplicación de una nueva cubierta. Estos procedimientos, aunque esenciales para el tratamiento, se describen a menudo como momentos de importantes experiencias dolorosas^(6,8)^.

Al considerar el contexto multifactorial y subjetivo, cabe destacar que el objetivo de la atención de enfermería para el manejo clínico del dolor no siempre será la analgesia, dada la naturaleza crónica de las heridas, y puede constituir mínimamente la promoción del confort, definido por Katharine Kolcaba como una experiencia inmediata de fortalecimiento a partir de las necesidades de alivio, tranquilidad o trascendencia, que se satisfacen en los contextos físicos (en referencia a las sensaciones corporales), psicoespirituales (significado de la vida/espiritualidad, lo que incluye la autoestima y el autoconcepto), ambientales (relacionados con los elementos del entorno) y socioculturales (que implican relaciones interpersonales, familiares, sociales y económicas)^(9,10)^.

La literatura reúne revisiones que describen intervenciones farmacológicas y no farmacológicas para el manejo del dolor en el contexto de heridas complejas, con avances en tecnologías, estrategias analgésicas y enfoques complementarios, como técnicas de relajación, musicoterapia y terapia cognitivo-conductual^(11,12,13,14)^. Sin embargo, estas publicaciones, aunque relevantes, tienen un enfoque predominantemente clínico y biomédico, sin organizar la atención desde una perspectiva ampliada que contemple las dimensiones del confort del paciente.

Por lo tanto, se observa una laguna en la investigación que justifica el desarrollo y la sistematización de intervenciones basadas en referencias teóricas de enfermería, en especial en la Teoría del Confort de Kolcaba. Este marco, al comprender el confort como un resultado medible de la atención y como un instrumento que orienta el proceso de trabajo de la enfermería, permite un enfoque sistematizado, holístico y centrado en el paciente.

A partir de estas consideraciones, el presente estudio tuvo como objetivo mapear la evidencia científica de las intervenciones para el manejo del dolor en personas con heridas complejas, desde la perspectiva de la teoría del confort de Kolcaba.

## MÉTODO

### Tipo de Estudio

Se desarrolló un estudio de revisión, de tipo escopo, realizado de acuerdo con las directrices metodológicas de JBI para la revisión de escopo y de acuerdo con las recomendaciones de la directriz *Preferred Reporting Items for Systematic Reviews and Meta-Analyses extension for Scoping Reviews* (PRISMAScR)^(15,16)^. El protocolo de revisión se registró en el Open Science Framework (OSF) con el DOI 10.17605/OSF.IO/Q3B25 para hacer pública la información del estudio, fomentar la transparencia y permitir que se replique la estrategia de búsqueda de la investigación.

La revisión se desarrolló en las siguientes etapas: identificación de la pregunta de investigación, identificación de estudios relevantes, selección del estudio, mapeo de datos, recolección, resumen y presentación de resultados^(17)^.

### Pregunta de Investigación

Se utilizó la estrategia mnemónica Población, Concepto y Contexto (PCC), siendo Población: adultos con heridas complejas; Concepto: intervenciones realizadas para el manejo del dolor; y Contexto: servicio de atención de salud, lo que orientó la elaboración de la pregunta guía de la revisión: “¿Qué evidencia científica hay disponible en la literatura sobre las intervenciones para el manejo del dolor en personas con heridas complejas, desarrolladas en los servicios de salud?”. La búsqueda se realizó entre diciembre de 2024 y abril de 2025.

### Criterios de Elegibilidad

Para la selección del material que compuso el corpus textual, se adoptaron como criterios de inclusión: artículos originales completos, disponibles íntegramente y de forma gratuita en formato electrónico (acceso CAFe); disertaciones y tesis, sin restricción de idiomas y sin límite temporal. Los criterios de exclusión fueron: cartas al editor y cartas al lector, resúmenes en actas de eventos, artículos incompletos, estudios en fase de proyecto o aún sin resultados.

### Estrategia de Búsqueda

Se realizó una búsqueda en las bases de datos *National Library of Medicine and National Institutes of Health* (PUBMED), *Web of Science* (WOS), *Excerpta Medica dataBASE* (Embase), *Cumulative Index to Nursing and Allied Health Literature* (CINAHL), SCOPUS (Elsevier), *Cochrane Library*, Literatura Latinoamericana y del Caribe en Ciencias de la Salud (LILACS), a las que se accedió a través de la Comunidad Académica Federada (CAFe), utilizando términos que figuran en *Medical Subject Headings* (MeSH) y Descriptores en Ciencias de la Salud (DeCS). La revisión también abarcó la literatura no convencional (literatura gris) del repositorio ProQuest Dissertations and Thesis Global, Biblioteca Digital Brasileira de Teses e Dissertações (BDTD) y Google Scholar (limitado a los 100 primeros resultados).

Los términos clave utilizados fueron “manejo del dolor”, “heridas y lesiones”, “dolor crónico”, “protocolos clínicos” y “analgesia”. Para aumentar la sensibilidad de la búsqueda, estos términos se combinaron con sus traducciones correspondientes, como “*Pain Management*”, “*Wounds and Injuries*”, “*Chronic Pain*”, “*Clinical Protocols*” y “*Analgesia*”; se utilizaron los operadores booleanos OR y AND. Se desarrolló una estrategia de búsqueda específica para cada base de datos, respetando sus particularidades, con el objetivo de optimizar la recuperación de información relevante, tal y como se describe en la Cuadro 1.

**Cuadro 1:** Estrategias de búsqueda utilizadas en las bases de datos – João Pessoa, PB, Brasil, 2025.

Base de datos	Estrategia de búsqueda
MEDLINE/PubMed	(“Pain Management”[MeSH Terms] OR “Pain Management”[Title/Abstract]) AND (“Wounds and Injuries”[MeSH Terms] OR “Wounds and Injuries”[Title/Abstract]) AND (“Chronic Pain”[MeSH Terms] OR “Chronic Pain”[Title/Abstract])
Embase	(‘complex wounds’ OR ‘chronic wounds’:ti,ab,kw OR ‘chronic wounds’) AND (‘pain management’:ti,ab,kw OR ‘pain management’) AND (‘analgesia’:ti,ab,kw OR ‘analgesia’)
SCOPUS	(TITLE-ABS-KEY (“Complex Wounds”) OR TITLE-ABS-KEY (“Wounds and Injuries”) AND TITLE-ABS-KEY (“Pain Management”) AND TITLE-ABS-KEY (“Chronic Pain”))
*Web of Science*	TS = (“Pain management”) AND TS = (“Wounds and injuries”) OR TS = (“Complex Wounds”) OR TS = (“Chronic Wounds”) AND TS = (“Chronic Pain”) AND TS = (“Clinical Protocols”) OR TS = (“Treatment Protocols”)
LILACS	(“Manejo da Dor”) OR (“Manejo del Dolor”) AND (“Ferida”) OR (“Herida”) OR (“Feridas”) OR (“Heridas”) AND (“Dor Crônica”) OR (“Dolor Crónico”) AND (“Cuidados de Enfermagem”) OR (“Atención de Enfermería”) OR (“Assistência de Enfermagem”) OR (“Cuidado de Enfermería”) AND (instance:”regional”)
*Cochrane Library*	“Pain Management” in Title Abstract Keyword AND “Wounds and Injuries” in Title Abstract Keyword OR “Complex Wounds” in Title Abstract Keyword OR Injury in Title Abstract Keyword AND “Chronic Pain” in Title Abstract Keyword
CINAHL	“Pain Management” AND (“Wounds and Injuries”) OR Injury AND “Chronic Pain” AND “Clinical Protocols”
Biblioteca Digital Brasileira de Teses e Dissertações	Feridas AND “Manejo da Dor”
ProQuest	(“Complex Wounds” OR “Chronic Wounds”) AND “Chronic Pain”
Google Scholar	(“Complex Wounds” OR “Chronic Wounds” OR “Wounds and Injuries” OR Injury) AND “Pain Management” AND “Chronic Pain” OR “Analgesia”

Fuente: Datos de la investigación, 2025.

### Selección de los Estudios

Tras la búsqueda en las bases de datos, todos los registros encontrados se exportaron al gestor de referencias Zotero, versión 7.0.10, para eliminar los duplicados. La selección y evaluación de los estudios fue realizada por dos revisores de forma independiente y ciega, recurriendo a un tercer revisor en caso de discrepancias, mediante la aplicación web Rayyan. La preselección de los estudios se realizó mediante la lectura del título y los resúmenes, basándose en los criterios de inclusión y exclusión. Los estudios preseleccionados se sometieron a una lectura completa para definir los elegibles.

Por último, se realizó una búsqueda en las listas de referencias de los estudios incluidos, con el fin de identificar otros artículos no localizados en búsquedas anteriores. Los estudios considerados elegibles en esta etapa se incluyeron a los demás previamente seleccionados y formaron parte del corpus de la revisión exploratoria. El proceso de selección se presenta según el diagrama de flujo de PRISMA 2020.

### Extracción de Datos

Para la extracción de la información del corpus se utilizó un instrumento elaborado por los investigadores, estructurado en una hoja de cálculo de Microsoft Excel, con las siguientes variables: base, revista, año de publicación, autoría, idioma, DOI, título, país de origen, objetivo, diseño metodológico, principales resultados y conclusión.

### Presentación de los Datos

Se elaboró una síntesis de los contenidos de cada estudio, dando lugar al corpus textual, que a su vez se sometió al software Interface de R pour les Analyses Multidimensionnelles de Textes et de Questionnaires (IRaMuTeQ) versión 0.7 alpha2, mediante el análisis multivariante de Clasificación Jerárquica Descendente (CJD), que permitió agrupar vocabularios similares en clases de segmentos textuales y presentar las relaciones semánticas mediante un dendrograma generado por el software^(18)^.

Tras la presentación de la CJD propuesta por el software, se procedió a la reorganización de las clases (5 clases) según el análisis de contenido basado en el marco teórico de Katharine Kolcaba^(10)^, lo que permitió la identificación de 2 categorías.

### Aspectos Éticos

Esta revisión de alcance utilizó exclusivamente datos secundarios disponibles públicamente y, por lo tanto, no requirió la aprobación del Comité de Ética en Investigación. Todas las etapas se llevaron a cabo de conformidad con los principios éticos y las directrices metodológicas aplicables, garantizando el respeto de los derechos de autor de las producciones analizadas.

## RESULTADOS

El corpus textual del estudio resultó de 27 artículos entre los 1577 encontrados en las bases de datos y repositorios, cuyo proceso de identificación, selección y elegibilidad se realizó de acuerdo con la Figura 1.

**Figura 1:** Diagrama de flujo de PRISMA para la selección de estudios y el proceso de inclusión en la revisión de alcance. João Pessoa, PB, Brasil, 2025.

Con el fin de facilitar la presentación, se optó por organizar los datos más relevantes de caracterización de los artículos en un cuadro (Cuadro 2).

**Cuadro 2:** Caracterización de los estudios – João Pessoa, PB, Brasil, 2025.

ID^*^	Autor/Año	Título	Objetivo
A1	Bechert y Abraham, 2009^(13)^	Pain management and wound care	Discutir la etiología del dolor y proporcionar al lector estrategias para tratar las heridas dolorosas una vez identificadas.
A2	Ffrench et al., 2023^(19)^	Systematic review of topical interventions for the management of pain in chronic wounds	Establecer una comprensión sólida del nivel de eficacia de las intervenciones tópicas disponibles para el tratamiento del dolor asociado a las heridas en personas con experiencia vivida de heridas crónicas.
A3	Guglielmo, 2024^(20)^	The ‘Surrounding the Dragon’ (Wei Ci) Acupuncture Technique: A Systematic Review	Determinar la eficacia basada en la evidencia de la antigua técnica de acupuntura de la medicina tradicional china (MTC) “Rodeando al dragón”.
A4	Healy et al., 2023^(21)^	Chronic wound-related pain, wound healing and the therapeutic potential of cannabinoids and endocannabinoid system modulation	Resumir de forma exhaustiva los hallazgos hasta la fecha sobre el potencial de los cannabinoides, los moduladores del sistema endocannabinoide y los medicamentos a base de cannabis para el tratamiento del dolor crónico relacionado con las heridas y su cicatrización.
A5	Mervis y Federman, 2018^(11)^	Pain Management in Patients with Chronic Wounds	Proporcionar una estructura basada en la evidencia para el tratamiento farmacológico y no farmacológico del dolor en pacientes con heridas crónicas.
A6	Metcalfe, 2015^(22)^	Pain management in palliative patients with chronic wounds	Definir el dolor en relación con las heridas crónicas paliativas, examinar la incidencia y las tasas de dolor crónico en este grupo de pacientes y analizar la literatura para determinar las mejores estrategias de gestión.
A7	Price et al., 2007^(12)^	Managing painful chronic wounds: the Wound Pain Management Model	Evaluar el dolor de la herida y las estrategias de tratamiento, centrándose en el dolor crónico persistente y el dolor asociado al tratamiento local de la herida, incluido el cambio de vendajes.
A8	Richardson, 2012^(23)^	An introduction to the biopsychosocial complexities of managing wound pain	Discutir el tratamiento de las heridas agudas y crónicas, centrándose en las habilidades específicas necesarias para manejar el dolor asociado a diferentes tipos de heridas y abordar las áreas importantes del cambio de vendajes.
A9	Woo et al., 2013^(24)^	Evidence-based approach to manage persistent wound-related pain	Proporcionar un enfoque basado en la evidencia para el tratamiento del dolor persistente en personas con heridas crónicas.
A10	Arapoglou et al., 2011^(25)^	Analgesic efficacy of ibuprofen-releasing foam dressing compared with local best practice for painful exuding wounds	Examinar si la etiología de la herida tiene algún efecto sobre las propiedades analgésicas de un apósito de espuma liberadora de ibuprofeno, que anteriormente ha demostrado reducir el dolor en heridas de diversas etiologías, en comparación con las mejores prácticas locales (LBP).
A11	Araújo et al., 2021^(8)^	Realidade virtual durante o manejo da dor em pessoas com feridas crônicas: estudo experimental	Evaluar el uso de la realidad virtual en entornos de inmersión y simulaciones realistas para el alivio del dolor en pacientes con heridas crónicas durante el cambio de vendajes.
A12	Gottrup et al., 2007^(26)^	Less pain with Biatain–Ibu: initial findings from a randomised, controlled, double-blind clinical investigation on painful venous leg ulcers	Investigar si el apósito de espuma con ibuprofeno alivia el dolor de la úlcera venosa sin comprometer las propiedades beneficiosas de la cicatrización húmeda de la herida, con un perfil de seguridad aceptable.
A13	Humbert et al., 2013^(27)^	Efficacy and safety of a gauze pad containing hyaluronic acid in treatment of leg ulcers of venous or mixed origin: a double-blind, randomised, controlled trial	Investigar la eficacia y seguridad de una compresa de gasa que contiene ácido hialurónico (AH) en el tratamiento local de úlceras venosas en las piernas, en comparación con su vehículo neutro.
A14	Pittler y Ernst, 2012^(28)^	Horse chestnut seed extract for chronic venous insufficiency	Revisar la eficacia y la seguridad del extracto oral de semilla de castaño de Indias (HCSE) frente al placebo o la terapia de referencia para el tratamiento de la IVC.
A15	Romanelli et al., 2009^(29)^	Ibuprofen slow-release foam dressing reduces wound pain in painful exuding wounds: preliminary findings from an international real-life study	Comparar el efecto de un apósito de espuma que libera ibuprofeno (Biatain Ibu ®) con las mejores prácticas locales en el tratamiento de heridas exudativas dolorosas.
A16	Kitala et al., 2022^(30)^	Eye-tracked computer games as a method for pain perception alleviation in chronic wound management	Evaluar la utilidad de los juegos con seguimiento ocular que distraen al paciente durante las actividades de tratamiento de heridas en una clínica quirúrgica ambulatoria y, por lo tanto, verificar si vale la pena introducir los rastreadores oculares en la rutina clínica diaria.
A17	Rook et al., 2019^(31)^	Mepilex® Border Flex — results of an observational study in German specialist wound care centres	Evaluar el tratamiento de diversos tipos de heridas exudativas con un apósito de espuma recubierto de silicona autoadhesivo.
A18	Nair, 2018^(32)^	Microcurrent as an adjunct therapy to accelerate chronic wound healing and reduce patient pain	Evaluar la eficacia de la microcorriente como terapia complementaria para reducir el tamaño de la herida y la puntuación del dolor en pacientes con heridas crónicas.
A19	Swift et al., 2024^(33)^	Taking the pain out of wound healing with microcurrent electrical stimulation therapy	Evaluar el uso de Accel-Heal Solo para reducir el dolor asociado a las heridas difíciles de cicatrizar y su impacto en la dependencia de analgésicos.
A20	Tang et al., 2023^(34)^	The associations between diode laser (810 nm) therapy and chronic wound healing and pain relief: Light into the chronic wound patient’s life	Determinar las asociaciones entre la terapia con láser de diodo y la curación de heridas crónicas o el alivio del dolor.
A21	Acevedo, 2020^(35)^	Successful treatment of painful chronic wounds with amniotic and umbilical cord tissue: A case series	No presenta.
A22	Bradbury et al., 2008^(36)^	Measuring outcomes with complex patients: an audit of the effect of Actiform Cool on painful wounds	Explorar el efecto de Actiform Cool en el dolor relacionado con la herida.
A23	Flanagan et al., 2006^(37)^	Case series investigating the experience of pain in patients with chronic venous leg ulcers treated with a foam dressing releasing ibuprofen	Perfil y características de las personas que utilizan el apósito de espuma con ibuprofeno.
A24	Freedman et al., 2004^(38)^	Practical treatment of pain in patients with chronic wounds: pathogenesis-guided management	Elaborar una propuesta general para el manejo del dolor basada en la corrección de las causas patológicas de la herida crónica.
A25	Hampton, 2007^(39)^	Chronic pain in wounds: a report on 11 case studies	Investigar la eficacia de la reducción del dolor y el impacto en la calidad de vida del apósito impregnado con ibuprofeno.
A26	Maida y Corban, 2017^(40)^	Topical Medical Cannabis: A New Treatment for Wound Pain—Three Cases of Pyoderma Gangrenosum	Evaluar la eficacia y la seguridad del tratamiento con cannabis medicinal tópico compuesto en aceite de girasol orgánico no modificado genéticamente en pacientes con pioderma gangrenoso.
A27	Eriksson et al., 2021^(41)^	Chronic wounds: Treatment consensus	Formular un consenso imparcial sobre el mejor tratamiento para las heridas crónicas.

Fuente: Elaborado por los autores, 2025.

*A: artículo, seguido del número secuencial.

Como se observa en la Cuadro 2, los estudios se publicaron entre 2004 y 2024, con una mayor incidencia de publicaciones en los años 2023, con tres estudios (11,2%), y en 2024, con dos estudios (7,40%). También se observó una frecuencia similar de dos estudios (7.40%) en los años 2020, 2018, 2013, 2012, 2009, 2008 y 2007, mientras que en los años 2022, 2021, 2019, 2017, 2015, 2011, 2006 y 2004 solo se presentó un estudio cada uno (3.70%).

En cuanto a los países de origen, destacaron el Reino Unido con seis (22,3%), seguido de cinco (18,5%) de los Estados Unidos de América. Canadá, China, Dinamarca e Irlanda contribuyeron con dos estudios cada uno (7,40%). Brasil, Malasia, Italia, Grecia, Francia, Polonia, Alemania e Inglaterra contribuyeron con un estudio cada uno (3,70%).

En cuanto a los niveles de evidencia, nueve (33,3%) eran de nivel I, seis (22,2%) de nivel II, dos (7,40%) de nivel III, tres (11,1%) de nivel V, seis (22,2%) de nivel VI y, por último, uno (3,70%) de nivel VII. En cuanto al idioma, todos los artículos estaban en inglés.

La Figura 2 ilustra el dendrograma resultante del análisis de CJD, compuesto por 5 clases denominadas según la expresión del contenido y procedentes del corpus textual, constituido por 27 textos, 116 segmentos textuales y 4072 palabras, cuyo índice de aprovechamiento fue del 79,31% de los segmentos procesados, lo que se considera un buen porcentaje de aprovechamiento.

**Figura 2:** Dendrograma de clasificación jerárquica descendente del*corpus*textual procesado por el software IRaMuTeQ. João Pessoa, PB, Brasil, 2025.

Tras el ejercicio analítico, respaldado por el marco teórico de Katharine Kolcaba^(10)^, sobre este corpus textual categorizado por el software IRaMuTeQ, surgieron dos categorías y cuatro subcategorías. Las categorías identificadas representan intervenciones físicas y psicoespirituales para el manejo del dolor en heridas complejas, que se presentan a continuación, en la Figura 3.

**Figura 3:** Categorización del corpus según las intervenciones para el manejo del dolor en personas con heridas complejas, basada en los contextos de la Teoría del Confort. João Pessoa, PB, Brasil, 2025.

### Categoría 1: Elementos del contexto físico del confort: instrumentos de medición del dolor físico y de la eficacia de las intervenciones; tecnologías y enfoques terapéuticos; y tratamientos farmacológicos para el alivio del dolor.

En la categoría 1, se destacó la dimensión física de la Teoría del Confort, que recomienda el uso de instrumentos, intervenciones y tecnologías que permitan medir y aliviar el dolor físico, favoreciendo respuestas terapéuticas más eficaces en el cuidado de personas con heridas complejas.

#### *Subcategoría 1* – Instrumentos de medición del dolor físico y de la eficacia de las intervenciones

La subcategoría 1 muestra los instrumentos de medición y su eficacia en las intervenciones para el manejo del dolor, como el uso de escalas estandarizadas, como la Escala Visual Analógica (EVA), que permite una evaluación objetiva del dolor, antes y después de la implementación de las intervenciones, al permitir verificar si hubo una reducción estadísticamente significativa de los síntomas algicos^(21,22,25,26,27,28,29,31,32,35,36,40)^.

#### *Subcategoría 2*– Resultado de las intervenciones para el alivio físico como parte del manejo del dolor

La subcategoría 2 refleja los resultados de las intervenciones físicas de cuidado directo de la herida como parte del manejo del dolor. Tras las acciones clínicas, los contenidos que se referían a los resultados eran: reducir, menor, bajo, mejorar, calidad y disminuir. Estas palabras reflejan la eficacia de las estrategias, como por ejemplo el uso de apósitos de gasa con ácido hialurónico (AH), que ha demostrado tener un impacto favorable en el dolor relacionado con la herida y en la calidad de vida de los pacientes^(27)^, y las técnicas de control del dolor^(26,28,35,36,37,39)^.

#### *Subcategoría 3*– Tecnologías y enfoques terapéuticos para el alivio del dolor físico

La subcategoría 3 abarca tratamientos específicos, tecnologías innovadoras y enfoques terapéuticos dirigidos directamente al manejo del dolor en heridas complejas. Entre los ejemplos, se destacaron la realidad virtual (RV) y el seguimiento ocular, ambos eficaces en la reducción de los niveles de dolor y de presión arterial. El tratamiento con láser de diodo también se describió como prometedor para acelerar la cicatrización de heridas crónicas y aliviar eficazmente el dolor en la herida, además de la terapia de estimulación nerviosa eléctrica transcutánea (TENS) y el uso de microcorrientes. Por último, se identificó el uso de tejidos derivados de la membrana amniótica (MA) y el cordón umbilical (CU) como una estrategia eficaz para la rápida reducción del dolor y la promoción del cierre de heridas crónicas e isquémicas dolorosas^(8,11,30,33,34,35)^.

#### *Subcategoría 4* – Tratamientos farmacológicos y tecnologías adyuvantes para promover el alivio del dolor

La subcategoría 4 aborda las terapias farmacológicas asociadas a otras tecnologías adyuvantes que, en conjunto, actúan en la promoción del cuidado físico. En este contexto, destacan las clases de anestésicos y analgésicos utilizados en el control del dolor, incluidos los antiinflamatorios no esteroideos (AINE), como el ibuprofeno, eficaces en el alivio del dolor en heridas cutáneas agudas y crónicas, especialmente cuando se presentan en apósitos de espuma^(22,24,25,26,29,37,39)^. Además, se planteó el uso de opioides, como el parche de liberación prolongada de fentanilo, utilizados en tratamientos para el dolor intenso^(38)^. El uso de cannabinoides ha demostrado su potencial para modular el comportamiento relacionado con el dolor inherente a las heridas^(21,40)^.

### Categoría 2: Elementos del contexto psicoespiritual del confort: Enfoques terapéuticos para el afrontamiento psicoespiritual.

En la categoría 2, se destacó la dimensión psicoespiritual de la Teoría del confort, cuyo contenido contempló intervenciones de cuidado físico y espiritual de la persona mediante enfoques terapéuticos complementarios o alternativos en el manejo del dolor. En esta categoría se destacaron como técnicas de relajación y terapias de eficacia probada la acupuntura, la terapia cognitivo-conductual, la meditación, la relajación muscular progresiva, la musicoterapia y el biofeedback^(11,13,20)^. Estas intervenciones representan el uso de técnicas no convencionales o complementarias como recursos valiosos en el control del dolor, alineándose con los principios de promoción del confort y de afrontamiento de los fenómenos intrínsecos a la convivencia con la herida y el dolor.

## DISCUSIÓN

Los resultados presentados en esta revisión de alcance han mapeado las intervenciones que se utilizan actualmente para el manejo del dolor en personas con heridas complejas y ofrecen una visión amplia de las estrategias adoptadas en la práctica clínica.

Las publicaciones presentaron diferentes intervenciones clínicas con impacto en la reducción del dolor asociado a las heridas complejas y, por lo tanto, en la promoción del alivio y el confort de las personas afectadas. Estos efectos favorecen la continuidad del tratamiento, al atender las necesidades físicas, en consonancia con los principios de la Teoría del Confort^(10)^. En este contexto, destaca la búsqueda de intervenciones que promuevan no solo el alivio del dolor físico, sino también la tranquilidad emocional y la trascendencia frente al sufrimiento, a fin de ampliar los efectos terapéuticos del cuidado de la herida en sí.

El uso de instrumentos de medición de calidad en la evaluación del dolor, como la EVA, ha demostrado ser esencial para medir de forma estandarizada y sensible la percepción del dolor en pacientes con heridas complejas. Los estudios realizados con el uso de la EVA han demostrado su utilidad en la identificación del dolor, su contribución a la sistematización de la atención personalizada y su mayor eficacia en la reducción de la intensidad del dolor, especialmente en grupos sometidos a intervenciones farmacológicas específicas, como el uso de apósitos impregnados con ibuprofeno^(21,22,25,26,27,28,29,30,31,32,35,36,40)^.

Entre las intervenciones analizadas, destacan los estudios que utilizaron apóses de espuma impregnados con ibuprofeno como una alternativa eficaz para el tratamiento de heridas dolorosas, los cuales han demostrado un alivio del dolor clínicamente significativo y han contribuido al confort del paciente durante el proceso de cicatrización^(24,25,26,29,37,39)^. El uso de estos apóses se configura como una forma de atender a la dimensión física del confort, un enfoque menos invasivo y potencialmente más seguro que los métodos sistémicos convencionales.

Además de los agentes tópicos, se ha observado la eficacia del uso de compresas de gasa que contienen ácido hialurónico. Los resultados indicaron efectos positivos en la reducción del dolor, la disminución del tamaño de la herida y la mejora de la calidad de vida de los usuarios, además de presentar un buen perfil de seguridad^(27)^. Estos hallazgos se ven corroborados por un estudio multicéntrico controlado^(42)^, que evaluó el uso de la misma intervención en úlceras de pierna de etiología venosa o mixta, y observó una reducción significativa del área de la herida, de la intensidad del dolor y una alta tasa de cicatrización completa.

La literatura reciente destaca el uso de tecnologías digitales emergentes, como la realidad virtual (RV), como estrategia complementaria a las prácticas analgésicas convencionales en el manejo del dolor físico. Las evidencias indican que la RV promueve un alivio inmediato y, en algunos casos, efectos prolongados incluso después de finalizar la inmersión. Además, el mantenimiento de la frecuencia cardíaca por debajo de 100 latidos por minuto durante su uso refuerza su seguridad^(8,11)^. Estos hallazgos sugieren que las tecnologías inmersivas e interactivas son herramientas con potencial para promover el confort en las dimensiones ambiental y psicoespiritual, al actuar sobre la distracción cognitiva, la reducción de la ansiedad y el manejo del dolor.

Paralelamente a las intervenciones de naturaleza digital, las terapias basadas en la luz, como el láser de baja intensidad, están ganando terreno en las prácticas asistenciales orientadas al dolor. Esta tecnología utiliza haces de luz concentrada, en el caso del láser de diodo, con una longitud de onda de 810 nm para estimular los procesos fisiológicos de cicatrización y promover efectos analgésicos, antiinflamatorios y reparadores. El estudio realizado en el Reino Unido demostró que este tipo de tratamiento no solo puede acelerar la cicatrización, sino también aliviar eficazmente el dolor asociado^(34,43)^. Aunque la intervención se muestra prometedora para promover el confort en el manejo del dolor en heridas complejas, su implementación efectiva en la realidad brasileña requiere inversión en capacitación, infraestructura, protocolos locales e investigaciones sobre eficacia y costo.

Asimismo, cabe destacar la aplicación de la estimulación eléctrica transcutánea y la microcorriente, como intervenciones con efectos beneficiosos tanto en la modulación del dolor como en la cicatrización de heridas difíciles de tratar. La terapia Accel-Heal Solo, basada en la estimulación eléctrica pulsada aplicada por electrodos colocados lejos de la herida, ha demostrado ser eficaz en el control del dolor y el edema y en la disminución de la necesidad de analgésicos locales o sistémicos^(33)^. De manera similar, la combinación de microcorriente con el tratamiento estándar dio como resultado una reducción significativa del tamaño de las heridas y de los niveles de dolor en todos los participantes, incluyendo la suspensión del uso de tramadol y la mejora de la calidad del sueño^(32)^.

Aunque todavía hay un número limitado de estudios sobre la aplicación de tejidos biológicos, como la AM, este enfoque ha demostrado su potencial para acelerar la cicatrización de heridas crónicas y promover el confort terapéutico. Este efecto puede atribuirse a las características naturales de la membrana avascular compuesta por colágeno, matriz extracelular natural y células madre^(35)^. Estos hallazgos refuerzan el valor clínico potencial de estos biomateriales como alternativa adyuvante, aún poco explorada.

Además de las estrategias no farmacológicas exploradas, los recursos farmacológicos como los AINE, los opioides y los anestésicos locales constituyen un enfoque esencial para el alivio del dolor, especialmente en casos de intensidad moderada a intensa; estos pueden administrarse por diferentes vías, según la naturaleza y las características del dolor. La elección adecuada de la clase y la vía de administración influye directamente en la eficacia terapéutica y la experiencia del proceso de cicatrización^(38,41)^.

Esto refuerza la idea de que la administración racional e individualizada de medicamentos es crucial para el control del dolor en heridas complejas. En este contexto, la existencia de protocolos basados en la evidencia puede orientar las decisiones terapéuticas en la atención de enfermería e instruir planes de atención más eficaces y orientados a las necesidades de confort autopercibidas.

Además de las formulaciones sistémicas, las intervenciones tópicas con analgésicos han demostrado ser eficaces para promover el confort y el alivio del dolor en las heridas crónicas. Los geles de morfina y diamorfina, por ejemplo, han demostrado proporcionar alivio durante hasta 24 horas, lo que contribuye a mantener el confort entre los intervalos entre un apósito y otro^(22,44)^. De manera similar, el uso de lidocaína al 2,75%, asociada al óxido de zinc, ha demostrado ser una alternativa rápida y duradera para el control del dolor, con efectos que se prolongan hasta cuatro horas, siendo especialmente útil en contextos de cuidados paliativos ambulatorios^(45)^. Estos hallazgos refuerzan el valor de la aplicación tópica de fármacos como estrategia adyuvante en el manejo del dolor de las heridas, con un impacto directo en la experiencia de confort de los pacientes.

Otro enfoque relevante fue el uso de parches transdérmicos de fentanilo, que permitieron la liberación continua y controlada del opioide, manteniendo niveles analgésicos estables en la sangre y reduciendo la aparición de efectos secundarios^(38)^. De manera complementaria, también se identificaron terapias alternativas como el cannabis medicinal tópico. Estudios de casos y evidencias recientes indican que la modulación del sistema endocannabinoide, mediante la aplicación tópica de aceite de cannabis y el uso de opioides, presenta potencial analgésico en heridas crónicas y pioderma gangrenoso, con reducción del dolor_(21,40)_.

En el contexto físico del confort, las intervenciones fitoterapéuticas también han demostrado su eficacia. Las evidencias apuntan a que el extracto de semilla de castaño de Indias (HCSE) promovió una reducción estadísticamente significativa del dolor en las piernas en comparación con el placebo. Este efecto se atribuye a las propiedades antiinflamatorias y venotónicas del extracto, que ayudan a disminuir el edema, mejoran la circulación sanguínea y favorecen la cicatrización^(28)^. De este modo, el HCSE se consideró una alternativa complementaria para el alivio del dolor y la promoción del confort físico en personas con úlceras venosas y otras heridas crónicas.

Después de explorar las estrategias orientadas al alivio del dolor físico, es necesario ampliar la mirada a otras dimensiones igualmente relevantes en el proceso de cuidado. En este contexto, el confort psicoespiritual^(10)^ implica afrontar las emociones negativas, la ansiedad, la desesperanza y recuperar el sentido de la vida, aspectos que influyen directamente en la recuperación y la calidad de vida. Las intervenciones dirigidas a esta esfera contribuyen a promover el bienestar general del paciente.

En esta dimensión, se destacó la técnica de acupuntura “*Surrounding the Dragon*” (SD), cuya eficacia se ha demostrado en el tratamiento de diversas afecciones dermatológicas, como úlceras por presión, herpes zóster, vitíligo no segmentario y envejecimiento de la piel^(19)^. Este enfoque se alinea con las prácticas de atención integral, para promover no solo beneficios físicos, sino también relajación, sensación de bienestar y equilibrio energético, fenómenos que intervienen en las experiencias algésicas de los individuos.

Otras estrategias ampliamente utilizadas en el manejo del dolor y la promoción del confort psicoespiritual fueron la terapia cognitivo-conductual, la atención plena, la meditación, la relajación muscular progresiva y el biofeedback^(11)^. Estas técnicas tienen un efecto reconocido en la reducción de la ansiedad, la regulación emocional y la resignificación de la experiencia dolorosa, aspectos directamente relacionados con el dominio psicoespiritual de la teoría.

Las intervenciones identificadas incluyen tecnologías como la realidad virtual, la terapia con láser y el cannabis medicinal, que se perfilan como estrategias innovadoras y potencialmente aplicables al contexto brasileño, sobre todo en los servicios ambulatorios^(8,46,47,48)^. Se trata de tecnologías viables y en expansión en el país, alineadas con las tendencias contemporáneas de atención y capaces de mejorar el manejo del dolor en personas con heridas complejas.

Entre las limitaciones de esta revisión, hubo dificultad en identificar intervenciones que contemplaran todos los dominios de la Teoría del Confort de Kolcaba, con predominio de los enfoques orientados a los contextos físico y psicoespiritual. Esta limitación puede atribuirse al escaso uso explícito de la teoría como referencia en los estudios, a las limitaciones de la operacionalización de los ámbitos sociocultural y ambiental en la práctica clínica, y a la tendencia de la literatura a dar prioridad a intervenciones más objetivas y fácilmente medibles.

Además, hay un predominio de estudios internacionales y niveles de evidencia bajos en parte de la muestra, lo que limita su aplicación directa en la realidad brasileña. Sin embargo, esta perspectiva internacional puede representar una oportunidad para identificar y adaptar estrategias exitosas y contribuir a la mejora de la asistencia en el contexto nacional, siempre que se respeten las particularidades locales.

## CONCLUSIÓN

El estudio permitió mapear las intervenciones que se utilizan actualmente en el manejo del dolor en personas con heridas complejas e indica que, sobre todo, se han implementado intervenciones físicas y farmacológicas que contribuyen a la experiencia inmediata de alivio y tranquilidad, tal como propone Kolcaba. Cabe destacar que también promovieron una mejora en la calidad de vida, favoreciendo la reducción del tamaño de las heridas y optimizando los procesos de cicatrización.

Sin embargo, dada la alta prevalencia del dolor asociado a las heridas complejas, llama la atención la escasez de intervenciones, especialmente en el contexto brasileño, que aborden específicamente el manejo del dolor en este público. Por lo tanto, el estudio proporciona fundamentos teóricos con potencial para instrumentalizar la atención profesional centrada en la persona, sensible a las dimensiones físicas y psicoespirituales de la Teoría del Confort de Kolcaba. En el contexto clínico, los hallazgos contribuyen a señalar posibilidades para decisiones de enfermería más asertivas y humanizadas, para fortalecer la implementación de prácticas basadas en la evidencia que, a través del alivio del dolor, también promueven el bienestar integral.

Así, la investigación se alinea con los principios de la Enfermería como ciencia del cuidado y responde a las demandas contemporáneas de producción científica que subsidian intervenciones más efectivas y humanísticas del cuidado de la persona y no solo el tratamiento de heridas de alta complejidad. En este sentido, se destaca la necesidad de desarrollar estudios nacionales que orienten la elaboración de protocolos de cuidado basados en la evidencia y fundamentados en referencias teóricas consistentes.

## Data Availability

Todo el conjunto de datos que apoya los resultados de este estudio fue publicado en el propio artículo.
